# Socioecology shapes child and adolescent time allocation in twelve hunter-gatherer and mixed-subsistence forager societies

**DOI:** 10.1038/s41598-022-12217-1

**Published:** 2022-05-16

**Authors:** Sheina Lew-Levy, Rachel Reckin, Stephen M. Kissler, Ilaria Pretelli, Adam H. Boyette, Alyssa N. Crittenden, Renée V. Hagen, Randall Haas, Karen L. Kramer, Jeremy Koster, Matthew J. O’Brien, Koji Sonoda, Todd A. Surovell, Jonathan Stieglitz, Bram Tucker, Noa Lavi, Kate Ellis-Davies, Helen E. Davis

**Affiliations:** 1 Department of Human Behavior, Ecology and Culture, Max Planck Institute for Evolutionary Anthropology, Deutscher Pl. 6, 04103 Leipzig, Germany; 2 Department of Comparative Cultural Psychology, Max Planck Institute for Evolutionary Anthropology, Deutscher Pl. 6, 04103 Leipzig, Germany; 3 Department of Archaeology, University of Cambridge, Downing Street, Cambridge, CV2 3DZ UK; 4 Department of Immunology and Infectious Diseases, Harvard T.H. Chan School of Public Health, 665 Huntington Ave, Boston, MA 02115 USA; 5 Department of Anthropology, University of Nevada, Las Vegas, 4505 S. Maryland Pkwy., Las Vegas, NV 89154 USA; 6 Department of Anthropology, University of California, Los Angeles, 375 Portola Plaza, Los Angeles, CA 90095 USA; 7 Department of Anthropology, Wayne State University, 656 W. Kirby St., 3037 FAB, Detroit, MI 48202 USA; 8 Department of Anthropology, University of Utah, 260 Central Campus Drive, Suite 4553, Salt Lake City, UT 84112 USA; 9 Department of Anthropology, University of Cincinnati, 481 Braunstein Hall, PO Box 210380, Cincinnati, OH 45221-0380 USA; 10 Department of Anthropology, California State University, Chico, 400 W. First St., Chico, CA 95929-0400 USA; 11 Faculty of Humanities and Social Sciences, Niigata University, 8050 Ikarashi 2-no-cho, Nishi-ku, Niigata, 950-2181 Japan; 12 Department of Anthropology, University of Wyoming, 12th and Lewis Streets, Laramie, WY 8207 USA; 13 Institute for Advanced Study in Toulouse, Université Toulouse 1 Capitole, 1 Esplanade de l’Université, 31080 Toulouse Cedex 06, France; 14 Department of Anthropology, University of Georgia, 250 Baldwin Hall, Athens, GA 30602 USA; 15 Department of Anthropology, University of Haifa, Abba Khoushy Ave 199, Mount Carmel, 3498838 Haifa, Israel; 16 Department of Anthropology, University College London, 14 Taviton Street, London, WC1H 0BW UK; 17 Department of Psychology, Swansea University, Singleton Park, Sketty, Swansea, SA2 8PP UK; 18 Department of Human Evolutionary Biology, Harvard, 11 Divinity Avenue, Cambridge, MA 02138 USA

**Keywords:** Human behaviour, Cultural evolution, Anthropology

## Abstract

A key issue distinguishing prominent evolutionary models of human life history is whether prolonged childhood evolved to facilitate learning in a skill- and strength-intensive foraging niche requiring high levels of cooperation. Considering the diversity of environments humans inhabit, children’s activities should also reflect local social and ecological opportunities and constraints. To better understand our species’ developmental plasticity, the present paper compiled a time allocation dataset for children and adolescents from twelve hunter-gatherer and mixed-subsistence forager societies (*n* = 690; 3–18 years; 52% girls). We investigated how environmental factors, local ecological risk, and men and women’s relative energetic contributions were associated with cross-cultural variation in child and adolescent time allocation to childcare, food production, domestic work, and play. Annual precipitation, annual mean temperature, and net primary productivity were not strongly associated with child and adolescent activity budgets. Increased risk of encounters with dangerous animals and dehydration negatively predicted time allocation to childcare and domestic work, but not food production. Gender differences in child and adolescent activity budgets were stronger in societies where men made greater direct contributions to food production than women. We interpret these findings as suggesting that children and their caregivers adjust their activities to facilitate the early acquisition of knowledge which helps children safely cooperate with adults in a range of social and ecological environments. These findings compel us to consider how childhood may have also evolved to facilitate flexible participation in productive activities in early life.

## Introduction

Human childhood is characterized by a longer period of parental provisioning and later sexual maturity than other great apes^1–4^. Our prolonged juvenile period is a heavily debated life history feature. Many scholars argue that long childhoods evolved as an extended period for learning how to exploit nutrient-dense and difficult-to-acquire food resources from our complex foraging niche, leading to higher productivity in adulthood^2,5^. Others argue that childhood is merely an artefact of our long lifespans^6,7^. A third perspective argues that juvenility is a transient helping stage in which children can make the best of growing slowly by leveraging their nonreproductive status into a higher reproductive potential for their mothers and indirectly for themselves^8–11^. Through the age-graded division of labour, children may specialize in tasks matched to their size in exchange for high-quality foods acquired by adults^12^. This intergenerational cooperation may have promoted a longer period of offspring dependence in our evolutionary history^1,12,13^.

Debates regarding the evolution of human life history have paid less attention to the considerable developmental plasticity evidenced in our species^9,14–17^. Human behavioural flexibility may have evolved to respond to the novel and unpredictable environments encountered during the evolution of the genus *Homo*^18^. This flexibility is reflected in the diverse cultural adaptions which enable humans to overcome challenges inherent to the range of environments we now inhabit^19,20^. If long childhoods maximize present and future production through learning and/or cooperation, then cross-cultural variation in these activities should reflect local social and ecological opportunities and constraints.

In the present paper, we investigated how socioecology affects child and adolescent activity budgets in twelve hunter-gatherer and mixed-subsistence forager societies—henceforth foraging societies. We focus on foraging societies because these were largely excluded from early comparative studies of childhood^21–23^ despite their relevance to understanding human evolution, diversity, and development^24,25^. To our knowledge, we have analyzed the largest available dataset on foraging child and adolescent time allocation, allowing us to systematically investigate cross-cultural correlates for child and adolescent behaviour in participating communities.

Previous research has demonstrated that children allocate time to a variety of cooperative and learning activities. While foraging children are not generally net producers, they can produce an energetic surplus when collecting resources that are abundant, easy, or safe to access^26–28^. In some cases, children’s food production can surpass their daily caloric needs^29–31^. Children provide childcare by carrying, holding, or playing with infants^13,32,33^, and participate in domestic chores such as food processing, water collecting, and cleaning^1,28,34–37^. These work activities may provide ‘on the job’ training^10,38–40^ and contribute to the socialization of gender roles^41–44^. Play makes up a large proportion of children’s time allocation^45^ and may contribute to adult skill acquisition^46^. Though the boundary between play and work is sometimes blurred^47^, children generally work more and play less as they age^46,48,49^. This suggests that play helps develop children’s cognitive and physical capacities^10,46,48,50–52^. Children’s play tends to mirror community-specific gender roles, thus contributing to the acquisition of gender-typical skills^46,53–55^. Considering this research, we focus our analyses on time allocation to food production, childcare, domestic work, and play.

Despite a long history of research on foraging children^24,56^, there is considerable unexplained cross-cultural variation in childhood time allocation to productive activities. In their survey of the Human Relations Area Files, Ember and Cunnar^57^ demonstrated that six- to ten-year-old children’s economic participation increased with subsistence strategy intensity, with foraging children working less, on average, than horticulturalist, intensive agriculturalist, and pastoralist children. Still, foraging children’s work was “the most variable—ranging from rare to substantial”. Variability in foraging children’s time allocation to food production, domestic work, and childcare was also highlighted in Kramer’s reviews of previously published data^15,58^. Anecdotal comparisons and population-specific studies of foraging childhoods, as well as comparative analyses of human behaviour more broadly, suggest that environmental factors, local ecological risk, and gendered division of food production labour may explain the observed cross-cultural variation in foraging children’s time allocation.

Environmental factors, such as climate and biomass, structure spatial and temporal resource distribution, which in turn shapes human subsistence strategies^59–61^. For example, hunter-gatherer residential mobility increases with primary biomass, reflecting lower resource availability in higher primary biomass environments^59^. Climate instability positively predicts subsistence diversification^62^. Communities living in harsher environments also exhibit more cooperative behaviours. Food and labour sharing is more extensive in communities that experience climate-related food-destroying natural hazards^63^. Alloparental care is more frequently observed in environments characterized by less predictable climates and lower average temperature and rainfall^64^. The age-graded division of labour, another important yet understudied form of cooperation^1^, may also be more pronounced in harsher environments. For instance, the Kalahari Desert is characterized by low water availability and habitat productivity. In this environment, San mother–child pairs had higher hourly caloric returns when children assisted with food processing in camp than when accompanying their mothers on foraging excursions^65^. Taken together, these findings suggests that in harsher environments—such as those with lower habitat productivity, water availability, and temperatures—children may specialize in childcare (i.e., act as alloparents) and domestic work^66^, thus freeing up stronger and more skilled co-residents to focus on food production^28,34,67^.

Children’s activities may also be affected by extrinsic risk of injury and/or mortality associated with their local ecologies. A series of studies conducted by Hawkes, Blurton Jones, and colleagues^65,68^ found that San children did not forage as frequently as Tanzanian Hadza children. While both communities lived in a savannah, water sources were more numerous and closer to food patches in Hadzaland than in the Kalahari. Hadzaland also had more landmark features. San children may thus have foraged less than Hadza children because the former faced higher risks of dehydration and getting lost. Foraging parents and children reported worrying about a variety of other ecological risks. For example, Hadza and Congolese BaYaka parents cited dangers associated with extreme weather, animal encounters, and travelling over challenging terrain as reasons not to forage with their children^69^. Bolivian Tsimane parents cited long distances and fear of environmental hazards (e.g., encounters with wild animals, dangerous terrain) as reasons to restrict children’s solo travel ranges^70^. Self-reported fear of getting lost constrained Hadza travel ranges until middle childhood^71^. In sum, children’s participation in activities that take them outside of settlements may be constrained by local ecological risk.

Finally, children’s social environments may also shape their time budgets. In all subsistence societies, men and women tend to target different foods. Generally, women tend to focus on more predictable and lower-risk resources (e.g., plants, fish), while men tend to focus on more variable and risky foods, such as large game, whose pursuit is often incompatible with the provisioning of high quality childcare^72–76^. However, this division of food production labour itself is highly variable across cultures^60^. In colder climates where fewer plant foods are available, women spend less time in direct food procurement, and instead, perform more domestic tasks while men primarily hunt^60,77^. Where plant foods are abundant and make up a large proportion of the diet, both men and women allocate time to gathering^60^, and women more frequently participate in hunting^78,79^. The gendered division of food production labour may be apparent in early ontogeny. For example, a comparison of Hadza and BaYaka forager children’s time allocation showed stronger gender differences in work and work-themed play among the former, potentially because Hadza adults maintain a more pronounced gendered division of food production labour than BaYaka^53,69^. Preferential emulation of same-gendered adults and active socialization via task assignment may help children learn community-specific adult gendered division of food production^42,44,80^, leading to cross-cultural differences in girls’ and boys’ time allocation.

**Figure 1:**
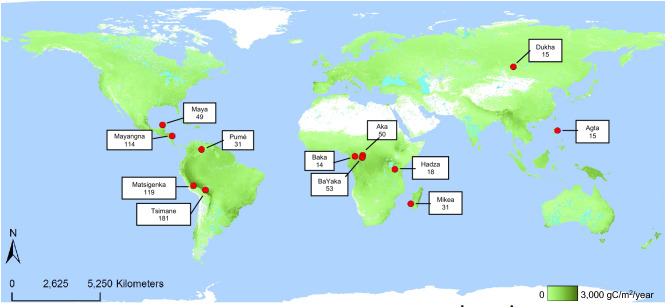
Map of the twelve study populations and worldwide Net Primary Productivity measured as gC/m^2^/year (2010 data; using MOD17A3, version-55, from the Numerical Terradynamic Simulation Group at the University of Montana^81^; map generated in ArcMap version 10.7^82^). Environments sampled in the present analysis ranged from rainforests to savannas. The resolution is 1 km. Sample sizes for each population are provided in the box (*N* = 690).

Considering the literature reviewed above, the present paper systematically investigated how cross-cultural variation in foraging child and adolescent time allocation was associated with environmental factors, ecological risk, and the adult gendered division of food production labour. To do so, we used a time allocation dataset comprised of observations for 690 children and adolescents (52% girls) ranging in age from approximately three to eighteen years inhabiting twelve foraging societies from Africa, Asia, Central America, and South America (Fig. 1). Using these data, we sought to answer three questions:

1. How do environmental factors influence child and adolescent time allocation? If communities living in harsher environments exhibit more cooperative behaviours^63,64^, then this association may also apply to the age-graded division of labour. Here, we estimated environmental harshness using three variables. Net Primary Productivity (NPP—gC/m^2^/year) measures the amount of new plant growth produced per year, reflecting habitat productivity. Annual Precipitation (mm) approximates total water inputs, reflecting water availability at the regional scale. Annual Mean Temperature (°C) approximates the total energy input for an ecosystem. We expected that child and adolescent participation in food production would be lower, and participation in childcare and domestic work higher, in harsher environments i.e., those with lower NPP, Annual Precipitation, and Annual Mean Temperature.
2. How does local ecological risk influence child and adolescent time allocation? Children’s activities are responsive to extrinsic risk of injury and/or mortality inherent to local ecologies^69–71^. Here, we estimated the effect of two extrinsic risk factors on time allocation. Dangerous Mammal Density (low/high) approximates the risk of predation or animal attack. Water Quality/Quantity ratings (four-point scale, 1 = high water quality/quantity) approximates the risk of dehydration and acute water-born illness at the local scale. We expected that children and adolescents would participate in less work in high-risk ecologies i.e., those with higher Dangerous Mammal Density and lower Water Quality/Quantity ratings.
3. How are gender differences in child and adolescent time allocation influenced by adult gendered division of food production labour? Boys’ and girls’ time budgets may reflect community-specific gendered labour divisions^53,69^. Here, we measured adult Gender Division of Food Production Labour as the estimated proportion that men and women contribute to overall daily caloric returns^60^ (standardized to between − 2 and 2). We expected that gender differences in children’s time allocation to work and play would be larger in societies with a more pronounced gendered division of food production labour.

## Results

### Descriptive statistics

Our sample includes 690 children and adolescents (52% girls) ranging in age from approximately three to eighteen years (*M* = 9.29 years, *SD* = 4.48). An average of 124.05 observations (*SD* = 160.88) were collected per child, totalling 85,597 unique observations. Fifty-five children from BaYaka, Dukha, Mikea, and Savanna Pumé datasets were sampled in more than one year. All other children were observed over a two to twelve months timespan during a single year. Please see the Supplementary Information (SI) for details regarding site-specific data collection procedures. Sampled communities practiced a wide range of subsistence strategies (Table 1), with reliance on non-foraged resources representing 5% (Hadza) to 97% (Matsigenka) of diets. While Fig. 1 shows that most participating communities inhabited tropical and subtropical climatic zones, there was considerable variation in NPP, annual mean temperature, and annual rainfall across our sample (Table 1).

**Table 1:** Sample characteristics and summary statistics.

Society	N children (sampled > 1 year)	% girls	Mean age (SD)^b^	Age range	Mean observations/child (SD)	Net primary productivity (gC/m^2^/year)	Annual mean temperature (°C)	Annual precipitation (mm)	Dangerous mammal density^c^	Water quality/quantity^d^	Proportion non-foraged food	Gendered division of food production labour^e^
Agta	15 (0)	33	6.13 (2.64)	3–12	21.27 (11.32)	1389.7	25.23	2653.69	Low	1	0.50	0.02
Aka	50 (0)	52	9.44 (3.89)	4–16	238.62 (53.49)	886.7	24.76	1551.03	High	1	0.49	− 0.08
Baka	14 (0)	50	9.21 (3.62)	5–15	720 (0)	1120	24.16	1570.26	High	1	0.30	− 0.15
BaYaka	53 (6)	42	11.02 (4.17)	3–18	253.87 (89.44)	969.6	24.81	1616.51	High	1	0.30	0.20
Dukha	15 (5)	53	9.17 (5.57)	3–18	577 (562.25)	142.4	− 6.71	411.20	Low	1	0.86	− 0.19
Hadza	18 (0)	78	8.39 (3.11)	3–14	35.72 (26.81)	601	21.44	673.74	High	2	0.05	0.18
Matsigenka	119 (0)	61	9.28 (4.62)	3–18	24.22 (11.64)	2438.6	17.71	834.53	Low	2	0.97	0.99
Maya	49 (0)	59	9.47 (4.98)	3–18	149.14 (16.69)	540	26.20	1058.72	Low	1	0.94	1.02
Mayangna	114 (0)	46	9.61 (4.89)	3–18	67.56 (17.86)	1220.5	25.89	2715.84	Low	1	0.77	0.99
Mikea^a^	31 (18)	48	11.51 (3.78)	6–20	150.13 (118.67)	1191.6	23.73	516.36	Low	4	0.45	− 0.02
Pumé	31 (26)	52	9.32 (4.41)	3–17	166.87 (81.44)	524.7	27.77	2069.46	Low	1	0.07	− 0.64
Tsimane	181 (0)	52	8.53 (4.30)	3–18	70.69 (20.76)	1952.5	26.11	1829.21	Low	2	0.77	0.43
Total	690 (55)	52	9.29 (4.48)	3–20	124.05 (160.88)	–	–	–	–	–	–	–

^a^Exact ages for Mikea children were not known. Children were instead categorized as early juveniles (5–8 years), late juveniles (9–15 years), and young adults (16–25 years). For this table, we held each category at the mean age; early juveniles = 6.5 years, late juveniles = 12 years, young adults = 20.5 years.

^b^The age of children with repeated observations was considered the mean of their age across all years sampled.

^c^Reflects the total density of dangerous mammals per km^2^ for each site. Low: n/km^2^ < 1. High: n/km^2^ > 10.

^d^1 = At the time of data collection, people usually or always had enough water and the water was of good quality, 2 = At the time of data collection, people usually or always had enough water, but the water was of poor quality, 3 = At the time of data collection, people rarely or never had enough water, but the water was of good quality, 4 = At the time of data collection, people rarely or never had enough water, and the water was of poor quality.

^e^Range: − 2, women do all the food production labour, 2, men do all the food production labour.

**Figure 2:**
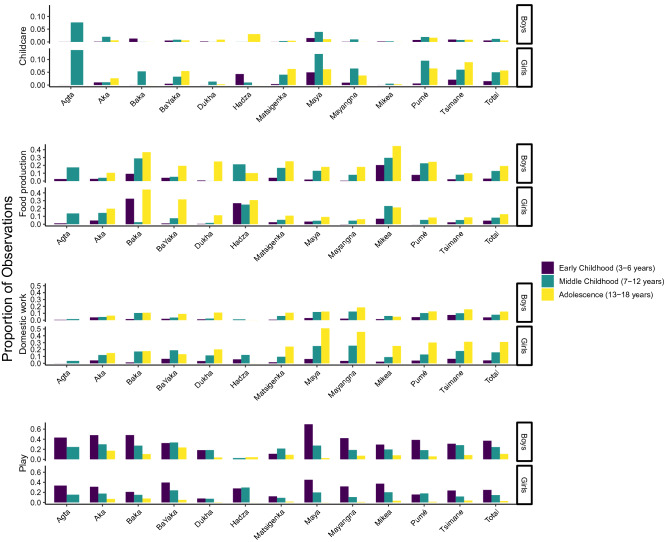
Time allocation by study population, gender, and age. Values represent mean individual proportion of total observed time.

Figure 2 provides a descriptive picture regarding cross-cultural variation in time allocation to work and play in early childhood (approx. 3–6 years), middle childhood (approx. 7–12 years), and adolescence (approx. 13–18 years) (see also Tables S2–S4). Across the sampled societies, the time children allocated to work was highly variable in middle childhood (childcare: 1–10% of observations; food production: 1–26%; domestic work: 3–20%). Compared to children in middle childhood, adolescents overall allocated more time to food production (21 vs. 11%) and household work (19 vs. 12%). Another robust trend was that across societies, boys’ participation in childcare was less than that of girls (1 vs. 4% overall, Table S4). Play represented a large proportion of children’s time allocation in early childhood (9–58%), after which participation in play decreased with age.

### Modeling strategy and model comparisons

We fit five Multilevel Multinomial Behavioural Models^83^ (MMBMs) to further examine foraging child and adolescent time allocation to play and work. Predictor variables for each model are presented in Table 2. In addition to the specific variables included in each model to assess the effect of environment, ecological risk, and gendered division of food production labour on children’s time allocation, all statistical analyses adjusted for the proportion of non-foraged foods consumed in each society. This is because previous studies have found that reliance on domesticated plants and animals is positively correlated with children’s time allocation to economic activities^57,84,85^. In what follows, we only discuss results pertaining to Models 3–5. Posterior means and standard deviations for the fixed effects in all five models can be found in Table 3. Note that in multinomial models, coefficients are not straightforward indicators for the effect of a variable on the probability of a behaviour^83^. We thus interpreted model estimates from Table 3 via predicted probabilities illustrated in the figures.

**Table 2:** Model summaries and comparison statistics.

Model	Predictor variables	WAIC (weight)
1—intercept only	None	176,083.7 (0.00)
2—individual-level effects	Age + gender + age x gender + prop non-foraged food	176,051.5 (0.01)
3—environmental factors	Model 2 + NPP + annual precipitation + annual mean temperature	176,052.3 (0.01)
4—ecological risk	Model 2 + dangerous mammal density + water quality/quantity	176,042.9 (0.81)
5—gendered division of food production labour	Model 2 + gendered division of food production labour + gendered division of food production labour x gender	176,045.9 (0.18)

All models include random effects for Individual and Society. *Age* Early Childhood (between 3 and 6 years of age, early juveniles for the Mikea dataset—reference category), Middle Childhood (between 7 and 12 years of age, late juveniles for the Mikea dataset), Adolescence (between 13 and 18 years of age, young adults for the Mikea dataset). Gender**:** Girls (reference category) and Boys. *Prop Non-Foraged Food* proportion of diet which was domesticated foods and foods purchased and/or traded. *Gendered Division of Food Production Labour* Standardized to between − 2, women do all the food production labour and 2, men do all the food production labour. *NPP* Net Primary Productivity in gC/m^2^/year. *Annual Precipitation* In mm. *Annual Mean Temperature* In °C. *Dangerous Mammal Density* Low/High. *Water Quality/Quantity* Four-point scale.

**Table 3:** Posterior means of fixed effects for models 1–5.

	Childcare	Food production	Domestic work	Play
**Model 1—intercept only**				
Intercept	** − 4.12 (0.53)**	** − 2.23 (0.33)**	** − 2.07 (0.23)**	** − 1.37 (0.21)**
**Model 2—individual-level effects**				
Intercept	** − 4.11 (0.69)**	** − 3.03 (0.34)**	** − 2.83 (0.26)**	** − 1.08 (0.22)**
Boys	** − 1.18 (0.28)**	0.05 (0.19)	− 0.12 (0.14)	**0.67 (0.11)**
Middle	**1.33 (0.23)**	**0.92 (0.14)**	**1.49 (0.12)**	** − 0.33 (0.10)**
Ado	**1.80 (0.23)**	**1.26 (0.17)**	**2.10 (0.13)**	** − 2.18 (0.15)**
Boys × middle	− 0.39 (0.34)	0.27 (0.22)	** − 0.66 (0.17)**	− 0.22 (0.14)
Boys × ado	** − 1.19 (0.39)**	**0.55 (0.25)**	** − 0.92 (0.19)**	**0.82 (0.19)**
Prop non-foraged^a^	0.09 (0.35)	− 0.45 (0.22)	0.09 (0.18)	− 0.13 (0.15)
**Model 3—environmental factors**				
Intercept	** − 4.00 (0.74)**	** − 3.16 (0.28)**	** − 2.92 (0.24)**	** − 1.19 (0.17)**
Boys	** − 1.18 (0.27)**	0.04 (0.19)	− 0.13 (0.14)	**0.66 (0.11)**
Middle	**1.34 (0.22)**	**0.93 (0.14)**	**1.50 (0.12)**	** − 0.32 (0.10)**
Ado	**1.80 (0.24)**	**1.27 (0.18)**	**2.10 (0.12)**	** − 2.18 (0.14)**
Boys × middle	− 0.42 (0.34)	0.28 (0.22)	** − 0.66 (0.17)**	− 0.22 (0.15)
Boys × ado	** − 1.18 (0.38)**	**0.55 (0.25)**	** − 0.90 (0.19)**	**0.83 (0.19)**
Prop non-foraged^a^	0.25 (0.43)	** − 0.42 (0.21)**	0.28 (0.19)	0.04 (0.15)
NPP^a^	− 0.25 (0.44)	0.07 (0.21)	− 0.26 (0.20)	− 0.16 (0.15)
Annual mean temp^a^	0.32 (0.56)	0.39 (0.28)	0.46 (0.26)	**0.50 (0.21)**
Annual prec^a^	0.28 (0.42)	− 0.38 (0.20)	− 0.05 (0.19)	− 0.03 (0.14)
**Model 4—ecological risk**				
Intercept	** − 2.61 (0.69)**	** − 2.96 (0.67)**	** − 2.18 (0.53)**	− 0.86 (0.42)
Boys	** − 1.16 (0.28)**	0.04 (0.19)	− 0.12 (0.14)	**0.68 (0.12)**
Middle	**1.38 (0.23)**	**0.93 (0.14)**	**1.50 (0.12)**	** − 0.32 (0.11)**
Ado	**1.85 (0.24)**	**1.27 (0.18)**	**2.11 (0.13)**	** − 2.16 (0.15)**
Boys × middle	− 0.43 (0.35)	0.28 (0.22)	** − 0.66 (0.17)**	− 0.23 (0.15)
Boys × ado	** − 1.22 (0.40)**	**0.55 (0.26)**	** − 0.91 (0.19)**	**0.80 (0.20)**
Prop non-foraged^a^	− 0.23 (0.33)	− 0.49 (0.25)	− 0.04 (0.23)	− 0.07 (0.19)
Mammal density	** − 1.37 (0.63)**	− 0.21 (0.52)	− 0.56 (0.49)	0.23 (0.40)
Water qual/quant	** − 1.07 (0.37)**	0.02 (0.33)	− 0.35 (0.26)	− 0.19 (0.21)
**Model 5—gendered division of food production labour**				
Intercept	** − 3.96 (0.75)**	** − 2.96 (0.38)**	** − 2.82 (0.35)**	** − 1.08 (0.26)**
Boys	** − 0.84 (0.31)**	− 0.09 (0.21)	− 0.03 (0.16)	**0.55 (0.13)**
Middle	**1.34 (0.24)**	**0.93 (0.14)**	**1.51 (0.12)**	** − 0.34 (0.10)**
Ado	**1.79 (0.24)**	**1.26 (0.18)**	**2.10 (0.13)**	** − 2.18 (0.15)**
Boys × middle	− 0.40 (0.35)	0.27 (0.22)	** − 0.67 (0.17)**	− 0.21 (0.15)
Boys × ado	** − 1.20 (0.39)**	**0.54 (0.25)**	** − 0.90 (0.19)**	**0.84 (0.20)**
Prop non-foraged^a^	0.20 (0.46)	− 0.45 (0.28)	0.11 (0.25)	− 0.21 (0.20)
Div	− 0.16 (0.77)	− 0.13 (0.57)	0.02 (0.52)	0.10 (0.44)
Div × boys	** − 0.86 (0.32)**	0.31 (0.23)	− 0.20 (0.15)	0.26 (0.16)

Standard deviations are in parentheses. Parameters in bold represent estimates whose 95% credible intervals do not cross zero.

*Boys* 1 = boys, 0 = girls. *Middle* 1 = Middle Childhood (between 7 and 12 years of age, late juveniles for the Mikea dataset), 0 = Early Childhood (between 3 and 6 years of age, early juveniles for the Mikea dataset). *Ado* 1 = Adolescence (between 13 and 18 years of age, young adults for the Mikea dataset). *Prop non-foraged* Proportion of domesticated foods and foods purchased and/or traded. *NPP* Net Primary Productivity in gC/m^2^/year. *Annual mean temp* Annual Mean Temperature, in °C. *Annual prec* Annual Precipitation, in mm. *Mammal density* Density of Dangerous Mammals, Low/High. *Water qual/quant* Water Quality/Quantity, four-point scale. *Div* Gendered Division of Food Production Labour, standardized to between − 2, women do all the food production labour and 2, men do all the food production labour.

^a^These values were *z*-score standardized.

Model 4 examining the effects of Ecological Risk on child and adolescent time allocation had the lowest Widely Applicable Information Criteria (WAIC), and 81% of the model weight, suggesting that this model had the highest probability of making the best predictions if supplied with new data^83^ (Tables 2, S13). Model 5 examining the effect of adult Gendered Division of Food Production Labour on girls’ and boys’ time allocation had 18% of the model weight. We note, however, that the standard errors for all models overlapped.

### Environmental factors

Model 3 examined the effect of environmental factors on child and adolescent time allocation. Contrary to our expectation, NPP, Annual Mean Temperature, and Annual Precipitation did not strongly predict child and adolescent participation in work. Figures S3–S5 shows that the effects of these environmental variables were relatively flat across activity categories. Annual Mean Temperature did positively predict child and adolescent participation in play (Fig. S4). Note however that only one study community (Dukha in Mongolia) had an Annual Mean Temperature below 0 (− 6.71 °C). The Annual Mean Temperature at all other sites was ≥ 17.71 °C. To investigate the possibility that a single study community disproportionally contributed to the effect of Annual Mean Temperature on play, we refit Model 3 without Dukha data. In this new model, the effect of Annual Mean Temperature on time allocation to play all but disappeared (Table S15, Fig. S8).

### Local ecological risk

**Figure 3:**
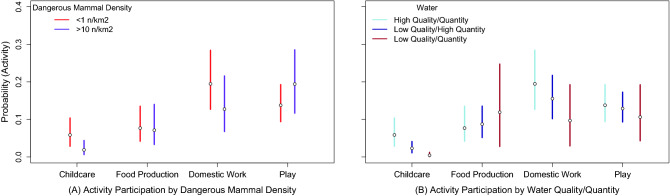
Model 4 predictions for the probability that a child engages in Childcare, Food Production, Domestic Work, and Play as a function of (**A**) Dangerous Mammal Density and (**B**) Water Quality/Quantity for children in middle childhood. Proportion Non-Foraged Food is held at the sample mean. Gender is held at the reference value (girls). Water Quality/Quantity and Dangerous Mammal Density are held at the reference value (low risk) in (**A**) and (**B**) respectively. Intervals represent 89th percentile credible intervals, as calculated from the posterior samples.

Model 4 examined the effect of local ecological risk on child and adolescent time allocation. In partial support for the expectation that risk would negatively affect participation in work, we found that Water Quality/Quantity and Dangerous Mammal Density negatively predicted child and adolescent participation in childcare and domestic work, though 95% Credible Intervals for the latter finding crossed zero reflecting estimate uncertainty. Figure 3 plots the predicted probabilities for activity participation for middle childhood. Higher Dangerous Mammal Density (Fig. 3A), and lower Water Quality/Quantity (Fig. 3B) negatively predicted participation in childcare and domestic work. However, risk had relatively little effect on participation in food production and play. Results from Models 3 and 4 were supported by those from additional analyses (see SI).

### Gendered division of food production labour

**Figure 4:**
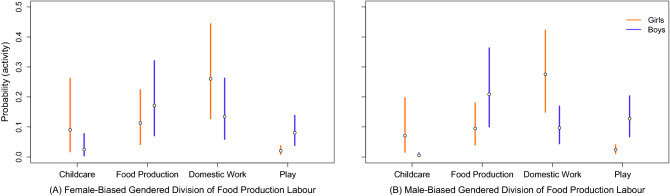
Model 5 predictions for the probability that adolescent girls and boys engage in Childcare, Food Production, Domestic Work, and Play in societies where (**A**) the Adult Gendered Division of Food Production Labour is biased towards women’s contributions (set at − 0.5) and (**B**) biased towards men’s contributions (set at 1). Proportion of Non-Foraged Food is held at the sample mean. Intervals represent 89th percentile credible intervals, as calculated from the posterior samples.

Model 5 examined the effect of adult Gender Division of Food Production Labour on girls’ and boys’ time allocation. In line with our expectations, gender differences in all activities were more pronounced in societies where men played a larger part than women in food production. However, only the effect of adult Gendered Division of Food Production Labour on gender differences in childcare was estimated with certainty (i.e., 95% Credible Interval did not cross zero). Figure 4 plots the predicted probabilities for adolescent girls’ and boys’ participation in childcare in societies that vary in gendered division of food production labour. This figure shows that boys’ participation in childcare is particularly low in societies where men played a larger part than women in food production (see also Fig. S7).

## Discussion

In this paper, we examined cross-cultural correlates for hunter-gatherer and mixed-subsistence forager child and adolescent time allocation. Previous anecdotal comparisons and population-specific studies of foraging childhoods, as well as comparative analyses of human behaviour more broadly, suggested that ecological and social patterns may explain variability in foraging children’s learning and cooperative activities. We empirically investigated these associations by examining how environmental factors, local ecological risk, and adult gendered division of food production labour influenced children’s participation in childcare, food production, domestic work, and play. We found (1) no support for an effect of environmental factors on childhood activities, (2) some support for the expected effect of local ecological risk on children’s work participation, and (3) that adult gendered division of food production labour predicted increased gender differences in child and adolescent time allocation, especially in childcare. In what follows, we discuss the implications and limitations of our findings, and point to avenues for future research.

We expected that children and adolescents in harsher environments would allocate less time to food production and more time to childcare and domestic work, reflecting a stronger age-graded division of labour. However, we found no strong effect of environmental factors on participation in these work activities. There are several potential reasons for this. First, sampled societies were heavily skewed towards tropical and subtropical regions. Though there is still considerable variation in sampled environments, only Dukha inhabit a northern latitude, reflecting a broader dearth of time allocation data from foraging societies inhabiting colder climates. Our findings may thus be explained by the limited variation in climates in our sample. Alternatively, parents and alloparents may buffer children from the direct effects of the environment through provisioning and care^64,86^. For example, parents and alloparents may teach children to navigate environmental challenges (e.g., extreme temperatures, resource scarcity), assign tasks that limit children’s exposure to potential environmental harms, and coordinate their labour with that of children to ensure safe participation across a range of economic activities. In other words, rather than dividing childcare, food production, and domestic work between children and adults, the age-graded division of labour may transect these activities such that children simultaneously learn to cope with various challenges inherent to their environments, contribute to the household economy, and gain ‘on the job’ training across a range of tasks. To test this possibility, future studies should investigate how household composition^22,34,83,87^ and co-resident activity^1,34^ affect child and caregiver time allocation in diverse environments.

Children in safer ecologies (i.e., high water quality/quantity, low dangerous mammal density) were more likely to participate in childcare and domestic work. Contrary to previous studies^65,88^, we found that children did not participate more in food production in safer ecologies. These findings may reflect activity-specific skill- and strength-based requirements. Kramer^1^ reports that many childcare and domestic work activities performed by children require relatively low strength and skill to complete. Safety may represent the main barrier to children’s participation in these activities. For example, children living in ecologies where drinking water can be found near camp may more often be tasked with fetching water than those for whom drinking water is very far. Parents may be less likely to entrust infant care responsibilities to children in ecologies where infants face greater risk of predation. Many food production activities (e.g., tuber digging, honey collecting, large game hunting) require higher strength and/or skill associated with locating resources and extracting them with specialized tools^1,14,89^. When children have developed the physical and cognitive capabilities needed to productively participate in food production, they also may have learned to safely navigate associated ecological risks. Further, risks associated with dangerous mammals are lessened when children accompany adults on foraging excursions than if they are in child-only groups^87^. Adults foraging with children are more likely to participate in food acquisition strategies that limit children’s exposure to risk^90,91^. When foraging in child-only groups, children may focus on collecting resources that are abundant closer to settlements or water sources in order to avoid hazards^70,88,92^. Where children cannot participate directly in food production, they may develop related skill and strength through play^46^. Because play usually occurs on the periphery of settlements^53,93–95^, this activity may be less directly affected by local ecological risk. In sum, social learning and social foraging may represent important mechanisms through which children adapt their foraging strategies to their local ecologies. Future studies should investigate how the composition of foraging groups and the size and distribution of children’s travel ranges maximize participation in food production activities while mitigating against exposure to local ecological risk.

Gender differences in all activity categories increased alongside men’s dietary contributions. This effect was strongest for participation in childcare. Alloparental care is prevalent in all human societies^96^, and children are important alloparents across cultures^13,32,97,98^. Still, how much alloparental care infants receive, and from whom, is highly variable^13,64,99^. Our findings suggest that, at least in foraging societies, boys’ participation in childcare is more context-dependent than that of girls. This may directly reflect fathers’ variable participation in direct infant care^100–102^. Where men play a larger role in food production, they may spend less time in direct infant caretaking^103–105^. In such cases, boys may have fewer opportunities to observe same-gender models in childcare, and thus, may be less inclined to participate in this activity^53,106^. Because task assignment contributes to the socialization of gender roles^21,42,44,66^, boys may also be given fewer childcare tasks in societies where such tasks are less normative for men. Taken together, these findings shed light on the ontogeny of the gendered division of food production labour by showing that differences in activity budgets are contingent on the social context in which children and adolescents grow up. Future studies should investigate how task assignment and autonomous participation in childcare vary alongside adult gendered division of food production labour. Disentangling these effects can elucidate the proximate mechanisms that contribute to gender differences in behaviour^42^, as well as how parents manipulate children’s time allocation according to the short-term and long-term needs of the household^50^.

### Limitations

There are several limitations to the present study. We pooled behavioural observations of foraging children and adolescents that were collected using different methods and across different temporal scales (see “Methods” and SI for details). The variability inherent to our data has limited our ability to make strong inferences. Further, our models most strongly predicted children’s participation in childcare, an activity that was variably defined across datasets, and relatively rare across societies. We also used broad behavioural categories to facilitate comparison across datasets. As a result, we were unable to investigate cross-cultural correlates for more nuanced activities (e.g., hunting vs. collecting). Environment may shape variation in children’s daily and seasonal activities rather than overall time allocation. Thus, our null findings should be interpreted cautiously. To facilitate comparisons of children’s direct and indirect provisioning, we focused on time allocation^9^. Children’s foraging returns, an alternative currency, may be differently influenced by environment and local ecological risk.

Our estimates for dangerous mammal density came from a global dataset^107^. Local-level densities would yield more precise results. Further, dangerous mammal density was split between sites in Africa (high) and all other sites (low) due to the risk posed by African elephants and hippopotami. Thus, it’s possible that these animals alone constrain children’s activities, or that other unmeasured geographical factors act as confounds within the model. Our measures for the gendered division of food production labour and the proportion of non-foraging foods consumed in each community were estimated by ethnographers. Ethnographer judgements have been used in other studies^108^, and many of us have collected foraging returns data in the surveyed communities, or have based our estimates on published values. In some cases, however, no contemporaneous empirical food return data exist. Thus, some estimates may be inaccurate, or may be biased by researcher gender, experience, or research methods.

Model comparisons are based only on the variables included in our analyses. They do not preclude the possibility that other variables outside the narrow range of social and ecological variables examined here might lead to models that garner more support^109^. While other environmental factors and local ecological risks may also affect children’s time allocation, the inclusion of additional variables may have saturated our models. The effects of additional environmental and ecological variables are reported in the SI. Further, the small sample of societies hindered our ability to account for potential interaction effects. For example, environment and ecological risk may predominantly constrain the time allocation of younger children, while adolescent activity patterns may more closely resemble those of adults. We note, however, that results from Models 3 and 4 remain consistent when excluding observations of adolescents (see SI for discussion).

Time allocation data often exhibits temporal autocorrelation, in the sense that observations taken closer together in time are more likely to evidence the same behaviour than those taken further apart^83,110^. In this study, the cross-cultural data were collected with heterogeneous sampling methods, and for some behaviors the temporal autocorrelation within study sites may be relatively high. In principle, modeling this autocorrelation is possible, though the cross-cultural data and multinomial outcomes in this study would require strong assumptions to be made at the outset. Therefore, we have elected not to impose autocorrelation structures on our models. We acknowledge that the parameter estimates may be biased as a result.

We did not account for children’s time allocation to school. This was because schooling was not available in all communities, with only four datasets having any observations for time allocation to school. The SI documents that, in half of the surveyed societies, there were no on-site schools. Even when school was available, ethnographers mostly report only sporadic attendance (see SI for details). Only among the Mayangna was school normatively compulsory and regularly attended. During the school year, schools may only run for a few hours a day, or may be closed due to teacher absence, inclement weather, or insufficient resources^111^. Children may also choose not to attend school, instead spending time with friends or in subsistence-based activities^111^. In some cases, children were primarily observed during the school holidays. Thus, observing children at school, or not, could be the result of several factors (e.g., school is not available, school is not in session, or the child is choosing not to attend school). This complexity complicated our ability to model time allocation to school, though this activity was included in as part of ‘other activities’ which acted as the reference category in the statistical models. We acknowledge that most observed children have spent some time at school, and that schooling is likely to impact children’s time allocation to work and play in some settings^112^.

## Conclusion

In this paper, we investigated how social and ecological environments shaped foraging child and adolescent time allocation. We did not find that child and adolescent participation in work and play was affected by environmental factors. We found that local ecological risk negatively predicted time allocated to childcare and domestic work, but not food production or play. Finally, we found that gender differences in child and adolescent activity budgets covaried with adult gender division of food production labour. We have argued that by coordinating labour by age and gender, children and adolescents may have ample opportunities to acquire skill—including through ‘on the job’ training, chore assignment, and teaching—which may help them overcome ecological risks and challenges. Strategies through which children and their caregivers adjust their behaviours to maximize safe cooperation in different socioecological settings may represent important cultural adaptations through which children learned to flexibly navigate novel environments encountered throughout our evolutionary history. In such contexts, learning would not only have provided deferred benefits in the form of higher adult foraging returns, but also immediate benefits to children by allowing them to safely participate in productive activities from an early age. We look forward to future empirical studies investigating the immediate and delayed benefits associated with knowledge acquisition across cultures, and modelling studies which formally consider how immediate and delayed learning benefits may have contributed to the evolution of long human childhood.

## Methods

This project was approved by the Simon Fraser University Office of Research Ethics (2020s0075). All data-contributing authors obtained ethical approval and/or in-country permission for data collection (see SI). All methods were performed in accordance with the relevant guidelines and regulations. All participants, parents, and/or legal guardians provided free, prior, and informed consent.

### Procedure

#### Behavioural observations

We pooled behavioural observational data collected in twelve societies that rely fully or partially on foraged resources for subsistence (Fig. 1; Table 1). Two behavioural observation techniques were used to collect the data: focal follow and scan sampling. Both methods are designed to systematically capture a representative sample of a population’s everyday life, allowing for quantitative analyses of time allocation. Focal follow sampling involves an observer recording a single individual’s activities during a specified period using predetermined behavioural coding schemes. During scan sampling, the observer records the activities of all individuals in a group at regular intervals^113^. Studies examining the validity and commensurability of these two methods have found that they provide comparable time budget estimates for non-human primates, though certain categories of behaviour (e.g., feeding time, social interactions) may diverge^114–117^. In humans, focal and scan sample data produced equivalent time budget estimates among Ache foragers^118^. Thus, the present paper considers data collected using focal and scan sampling methods as comparable.

Of the datasets included in the present study, four employed the focal follow sampling technique, with observations recorded continually or at one- to five-minute intervals. Three datasets scanned behaviour every 15–60 min within randomly allocated time blocks ranging from approximately 3–4 h. The remaining five datasets scanned behaviour daily, with observations ranging from every 2- to 3-min to 1–2 times a day. Some coding schemes included in the present analysis allowed for up to two categories to be coded simultaneously (e.g., activity coded as play and food production). Only 2.6% of observations from datasets which allowed for concurrent coding included activities which fell into two categories. We counted each of these observations as unique. Detailed descriptions regarding the setting and methods for data collection for each society can be found in the SI.

### Variables

#### Dependent variables

This paper focused on children’s participation in childcare, food production, domestic work, and play. Childcare was broadly defined as children tending to the physical or emotional needs of a young child or infant. Food production was defined as children’s tending to, or collecting, wild or domestic foods (e.g., gardening, hunting, etc.). Activities considered domestic work involved participation in household maintenance, such as food processing, cooking, and cleaning. Play was considered as consisting of intrinsically-motivated activities pursued for enjoyment^119^. To understand overall time allocation to each of these activities, we also accounted for other activities (e.g., socializing, eating, resting), which collectively acted as the reference category in the statistical models (see below). Table S1 describes the activities coded as childcare, food production, domestic work, play, and other activities for each dataset.

#### Age

Because many foraging societies do not record age in years^120^, exact ages could not be determined in all cases. Children were thus grouped into three developmental age categories: early childhood (between 3 and 6 years of age, early juveniles for the Mikea dataset), middle childhood (between 7 and 12 years of age, late juveniles for the Mikea dataset), and adolescence (between 13 and 18 years of age, young adults for the Mikea dataset).

#### Proportion of non-foraged foods

Each ethnographer estimated how many daily calories, on average, came from the following food categories for their field site: wild plant food, large game, small game, fish/seafood, insects, honey, domestic plants and animals, and traded/purchased foods. Where possible, ethnographers reported estimates based on published or unpublished food return data. Where contemporaneous data were not available, ethnographers estimated daily caloric returns based on their field experience. Because observations for Matsigenka were from published datasets^121,122^, we relied on values in Tables 14 and 16 in Johnson^123^. Values were reported as mean estimates, or as a minimum and maximum percentage. In the latter case, the dietary proportion produced for each food source in each society was calculated by taking the mean of the minimum and maximum reported dietary intakes per food category. All values were normalized so that the total intake from all sources for each society summed to 100%. The proportion of non-foraged foods was calculated by summing the proportion of domesticated foods and foods purchased and/or traded in each society.

#### Gendered division of food production labour

The degree to which men or women were the primary producers for each resource type was estimated by each ethnographer based on their field experience on a scale from 1 to 5 (1 = women only, 5 = men only). For Matsigenka, we relied on values published in Table 2 in Johnson^123^. Following Marlowe^60^, we estimated the Gendered Division of Food Production Labour by taking the average dietary proportion from each food source, multiplying it by the division of labour values, and summing across all food sources for each society. This yielded a Gendered Division of Food Production Labour statistic for each society that summarizes the extent to which either men or women contribute to overall daily caloric returns. We standardized this value to between − 2 (women do all the food production labour) and 2 (men do all the food production labour), with a value of 0 indicating that both genders contributed equally to food production.

#### Environmental factors

NPP (gC/m^2^/year) was calculated using data from the Net Primary Production dataset version-55 from the Numerical Terradynamic Simulation Group at the University of Montana^81^ (MOD17A3). This dataset is part of the NASA Earth Observation program, using Moderate Resolution Imaging Spectroradiometer satellite data. The dataset provides estimates of NPP at 1 km resolution for the earth’s entire land surface. We averaged the NPP values in the 9 square kilometers surrounding the location of each field site. We used the data layer from 2010 as it was consistently available for all field locations. Annual Mean Temperature and Annual Precipitation were calculated using the University of East Anglia Climate Research Unit gridded Time Series dataset^124^. This dataset, on a 0.5° latitude by 0.5° longitudinal grid, provides monthly climatic data spanning 1901–2018 interpolated using angular-distance weighting. Using the *raster*^125^ package in R^126^, we extracted maximum temperature (°C), minimum temperature (°C), and total monthly precipitation (mm) for each field site. An average of 3.48 and 6.42 weather stations contributed to the temperature and precipitation monthly values respectively. Monthly values were averaged over the 30 years preceding and including the most recent year of behavioural data collection for each field site. We then used these monthly values to calculate Annual Mean Temperature and Annual Precipitation using the biovars function in the *dismo*^127^ package. The specific formulas used for each variable can be found in O’Donnell and Ignizio^128^.

#### Local ecological risk

Dangerous Mammal Density was extracted from the PanTHERIA Database^107^ as follows: using maximum and minimum latitude and longitude values for all species in the database, we extracted a list of dangerous mammals (elephants, hippopotami, and carnivores over 50 kg) for each field site. We focused on mammals that could predate on children or that could attack children upon accidental encounter rather than mammals which may injure in self-defense, such as during hunting. Each ethnographer then reviewed the list for accuracy. Animals which were known to pose a risk at each site, but which were not reflected on the site-specific list, were added, including mammals under 50 kg (e.g., wolves). Mammals which appeared on the site-specific lists which were not known at the site were removed. Using the finalized list, we summed each species’ density (Pantheria variable *X21.1_PopulationDensity_n.km2*) to obtain a final value of the total density of dangerous mammals per km^2^ for each site. These values were naturally split between n/km^2^ < 1, and n/km^2^ > 10, the latter reflecting the high density of elephants and hippopotami, among other species, at African sites. We thus binarized Dangerous Mammal Density into low and high. Lists of Dangerous Mammals identified for each site can be found in Table S5. Water Quality/Quantity was measured using a cross-culturally validated four-point scale^129,130^. Each ethnographer rated their field site as follows: 1 = At the time of data collection, people usually or always had enough water and the water was of good quality, 2 = At the time of data collection, people usually or always had enough water, but the water was of poor quality, 3 = At the time of data collection, people rarely or never had enough water, but the water was of good quality, 4 = At the time of data collection, people rarely or never had enough water, and the water was of poor quality. No field sites were rated as 3. We were able to obtain inter-coder reliability for 8 of 12 field sites, for which there was 100% agreement between the ethnographers and the inter-coder.

### Statistical analysis

To identify cross-cultural correlates in child and adolescent activity, we followed Koster and McElreath^83^ in implementing MMBMs. MMBMs are multilevel multinomial logistic regressions, which can model categorical outcome variables while also accounting for repeated observations of individuals.

#### Model 1—intercept only

In this model, $K$ discrete activities follow a categorical (generalized Bernoulli) distribution for which the probability of observing each activity category $k$ is ${\uppi }_{\mathrm{k}}$. One activity category serves as the reference category to all other activities. Thus, the model is composed of $K-1$ equations that contrast the odds of performing activity $k$ instead of the reference category. We allowed the probabilities of performing activity $k$ to vary across individuals and societies. Random effects were added to each sub-equation to allow for individuals and societies to have greater or lesser odds of being observed in activity $k$ rather than the reference category. Our models include five activity categories ($k\in \{\mathrm{1,2},\mathrm{3,4}\}$ namely childcare, food production, domestic work, play—Table S1). The last category ($k=5,$ other activities) serves as the reference category. For each observation, the log-odds of individual *i* or society *j* performing childcare, food production, domestic work, or play instead of other activities is given by

1 $$\log \left( {\frac{{\pi_{kij} }}{{\pi_{5ij} }}} \right) = \beta_{k} + \nu \_individual_{ki} + \nu \_society_{kj} ;\;k \in \left\{ {1,\;2,\;3,\;4} \right\}$$

2 $$\left( {\nu \_individual_{1i} ,\nu \_individual_{2i} ,\nu \_individual_{3i} ,\nu \_individual_{4i} } \right)\sim {\text{Normal(0,}}\Omega_{I} {)}$$

3 $$\Omega_{I} = \left[ {\begin{array}{*{20}l} {\sigma_{{\nu \_individual_{1} }}^{2} } \hfill & {\sigma_{{\nu \_individual_{12} }} } \hfill & {\sigma_{{\nu \_individual_{13} }} } \hfill & {\sigma_{{\nu \_individual_{14} }} } \hfill \\ \; \hfill & {\sigma_{{\nu \_individual_{2} }}^{2} } \hfill & {\sigma_{{\nu \_individual_{23} }} } \hfill & {\sigma_{{\nu \_individual_{24} }} } \hfill \\ \; \hfill & \; \hfill & {\sigma_{{\nu \_individual_{3} }}^{2} } \hfill & {\sigma_{{\nu \_individual_{34} }} } \hfill \\ \; \hfill & \; \hfill & \; \hfill & {\sigma_{{\nu \_individual_{4} }}^{2} } \hfill \\ \end{array} } \right]$$

4 $$\left( {\nu \_society_{1j} ,\nu \_society_{2j} ,\nu \_society_{3j} ,\nu \_society_{4j} } \right)\sim {\text{Normal(0,}}\Omega_{S} {)}$$

5 $$\Omega_{S} = \left[ {\begin{array}{*{20}l} {\sigma_{{\nu \_society_{1} }}^{2} } \hfill & {\sigma_{{\nu \_society_{12} }} } \hfill & {\sigma_{{\nu \_society_{13} }} } \hfill & {\sigma_{{\nu \_society_{14} }} } \hfill \\ \; \hfill & {\sigma_{{\nu \_society_{2} }}^{2} } \hfill & {\sigma_{{\nu \_society_{23} }} } \hfill & {\sigma_{{\nu \_society_{24} }} } \hfill \\ \; \hfill & \; \hfill & {\sigma_{{\nu \_society_{3} }}^{2} } \hfill & {\sigma_{{\nu \_society_{34} }} } \hfill \\ \; \hfill & \; \hfill & \; \hfill & {\sigma_{{\nu \_society_{4} }}^{2} } \hfill \\ \end{array} } \right]$$

6 $$\sum\limits_{k = 1}^{K} {\pi_{kij} } \;\;\;\;\;\;{\text{for}}\;\;{\text{all}}\;\;i,\;j$$

where ${\beta }_{k}$ represents the intercepts that contrast activity $k$ with activity $k=5$. The individual-level random effects ${\upnu \_\mathrm{individual}}_{ki},k\in \{\mathrm{1,2},\mathrm{3,4}\}$ and the group-level random effects ${\upnu \_\mathrm{society}}_{kj}, k\in \{\mathrm{1,2},\mathrm{3,4}\}$ are assumed to be multivariate normally distributed with a zero mean and a homogenous (symmetric) 4 × 4 variance–covariance matrix. An individual-level varying intercept is positive $({\upnu \_\mathrm{individual}}_{ki}>0)$ when individual $i$ has an above average chance of performing activity $k$ instead of the reference activity, and vice versa. When the correlation between two behaviours is positive, an individual who participates more in the first activity also participates more in the second activity. When the correlation is negative, an individual who participates more in the first activity participates less in the second activity. Similarly, a societal-level intercept is positive (${\upnu \_\mathrm{society}}_{\mathrm{kj}}$ > 0) when an individual in that society has an above average chance of performing activity $k$ instead of the reference activity, and vice versa. The correlation between the random effects across activity categories (activity $k$ vs. activity $l, {\uprho }_{k,l}={\upsigma }_{\upnu \_\mathrm{individual}k,l}/{\upsigma }_{\upnu \_\mathrm{individual}k}{\upsigma }_{\upnu \_\mathrm{individual}l}$, and ${\uprho }_{k,l}={\upsigma }_{\upnu \_\mathrm{society}k,l}/{\upsigma }_{\upnu \_\mathrm{society}k}{\upsigma }_{\upnu \_\mathrm{society}l})$ are standardized to lie between – 1 and 1.

#### Model 2—individual characteristics

In addition to the individual and societal random effects presented in Model 1, Model 2 included individual-level variables for Age and Gender, as well as their interaction. We also adjusted for the Proportion of Non-Foraged Foods (*z*-transformed). Model 2 had the form

7 $$\log \left( {\frac{{\pi_{kij} }}{{\pi_{5ij} }}} \right) = \beta_{k} + \nu \_individual_{ki} + \nu \_society_{kj} + \sum\limits_{m} {\beta_{kim} x_{im} } \;\;\;\;\;\;k \in \left\{ {1,\;2,\;3,\;4} \right\}$$

where ${x}_{im}$ is the fixed effect $m$ that pertains to individual $i$ (age, gender, etc.) and the sums are over all fixed effects included in the model. The multivariate normal relationships between ${\upnu \_\mathrm{individual}}_{ki}$ and ${\upnu \_\mathrm{society}}_{kj}$, as shown in Eq. (2)–(5), remain the same, and we require all π_k_ to sum to 1 [Eq. (6)]. Models 3–5 build upon Model 2.

#### Model 3—environmental factors

To understand how environment influences children’s work and play, Model 3 included fixed effects for NPP, Annual Precipitation, and Annual Mean Temperature (all *z*-transformed).

#### Model 4—ecological risk

To understand how ecological risk influences children’s work and play, Model 4 included fixed effects for Dangerous Mammal Density, and Water Quality/Quality ratings.

#### Model 5—gendered division of food production labour

To understand how the gendered division of food production labour in adulthood influences gender differences in children’s work and play, Model 5 included a fixed effect of Gendered Division of Food Production Labour and an interaction for Gendered Division of Food Production Labour and Gender.

#### Estimation

The MMBMs were fit using the Hamilton Monte Carlo estimation implemented in *Rstan*^131^ and *rethinking*^132^. Non-centered parameterization of the varying effects using a Cholesky factorization of the variance–covariance matrices were relied upon^83^. We specified weakly informative priors for the fixed effect parameters and the variance–covariance matrices. Each model was run on 3 chains of 2000 iterations each. Half of these were discarded as warmup iterations. Model convergence was judged using the R-hat Gelman and Rubin convergence diagnostic. All R-hat values were smaller than 1.01, and there were no divergent iterations, suggesting good mixing across all models. Model fit was compared using WAIC. We report the means, standard deviations, and 95% credible intervals for the parameter estimates for all models. When plotting model predictions, we present 89% credible intervals which incorporate uncertainty in the fixed effect parameters.

## Supplementary Information

Supplementary Information.

## Data Availability

The Tsimane dataset is available upon reasonable request from Jonathan Stieglitz (jonathan.stieglitz@iast.fr). All other datasets and associated R scripts are available at https://github.com/sheinalewlevy/HGC-TA.
