# Supplementary material for: Socioecology shapes child and adolescent time allocation in twelve hunter-gatherer and mixed-subsistence forager societies
Source: Sci Rep. 2022 May 16;12:8054. doi: 10.1038/s41598-022-12217-1 (PMC9110336; doi:10.1038/s41598-022-12217-1)
Supplement: Supplementary file 1 — Supplementary Information. [file 41598_2022_12217_MOESM1_ESM.pdf]

Supplementary Information for: Socioecology shapes child and adolescent time allocation in twelve hunter-gatherer and mixed-subsistence forager societies

1. Site-specific ethnographic background and detailed data collection methods 4
1.1. Agta 4
1.2. Aka 4
1.3. Baka 5
1.4. BaYaka 6
1.5. Dukha 6
1.6. Hadza 7
1.7. Matsigenka 8
1.8. Maya 8
1.9. Mayangna 9
1.10. Mikea 10
1.11. Savanna Pumé 10
1.12. Tsimane 11
2. Supplementary Methods, Results, and Discussion 12
2.1. Correlation of random effects 12
2.2. Additional correlates for cross-cultural variation in time allocation 12
2.3. Assessment of multicollinearity and verification of model results 13
3. Supplementary Tables 14
Table S1. Description of activities coded as childcare, food production, domestic work, play, and other activities by sampled society. 15
Table S2. Time allocation by society, gender, and age. 16
Table S3. Time allocation by society and age. 18
Table S4. Time allocation by society and gender. 19
Table S5. Dangerous Mammal Species present at each site, and their associated density (n/km2). 20
Table S6. Medically Important Venomous Snake Species present at each site. 21
Table S7. Correlations and Variance Inflation Factors (VIF) for variables in Model 3. 23
Table S8. Correlations and Variance Inflation Factors (VIF) for variables in Model 4. 23
Table S9. Correlations and Variance Inflation Factors (VIF) for variables in Model 5. 23
Table S10. Correlation of individual-level and society-level random effects for Model 1 (intercept only). 24
Table S11. Correlation of individual-level and society-level random effects for Model 2 (Individual-level variables). 24

Table S12. Mean standard deviations of the random effects from the posterior samples for Models 1-5. 25

Table S13. Model comparisons for Models 1-5. 25

Table S14. Posterior means of fixed effects for supplementary models investigating each environmental factor and ecological risk effect independently. 26

Table S15. Posterior means of fixed effects for supplementary models investigating environmental factors and Annual Mean Temperature excluding observations from Dukha. 27

Table S16. Posterior means of fixed effects for Models 3 and 4 refit excluding data from adolescents. 28

Table S17. Posterior means of fixed effects for supplementary models investigating the effects of additional correlates for cross-cultural variation in time allocation. 29

4. Supplementary Figures 30

Figure S1. Correlation of individual random effects from Model 1. 30

Figure S2. Correlation of individual random effects from Model 2. 30

Figure S3. Model 3 predictions for Childcare, Food Production, Domestic Work, Play, and Other Activities (reference) as a function of Net Primary Productivity (NPP). 31

Figure S4. Model 3 predictions for Childcare, Food Production, Domestic Work, Play, and Other Activities (reference) as a function of Annual Mean Temperature. 32

Figure S5. Model 3 predictions for Childcare, Food Production, Domestic Work, Play, and Other Activities (reference) as a function of Annual Precipitation. 32

Figure S6. Model 4 predictions for the probability that a child engages in Childcare, Food Production, Domestic Work, Play, and Other Activities (reference) a function of (A) Dangerous Mammal Density and (B) Water Quality/Quantity for children in middle childhood. 33

Figure S7. Model 5 predictions for the probability children engage in Childcare, Food Production, Domestic Work, Play and Other Activities (reference) as a function of Gendered Division of Food Production Labour and Gender. 34

Figure S8. Model 3.5 predictions refitting Model 3 excluding the Dukha data for Childcare, Food Production, Domestic Work, Play, and Other Activities (reference) as a function of Annual Mean Temperature. 35

Figure S9. Model 3.4 predictions for Childcare, Food Production, Domestic Work, Play, and Other Activities (reference) as a function of Coefficient of Variation of Monthly Precipitation (CV %). 36

Figure S10. Model 4.3 predictions for Childcare, Food Production, Domestic Work, Play, and Other Activities (reference) as a function of total number of Medically Important Venomous Snake species. 37

Figure S11. Model 3.6 predictions refitting Model 3 excluding adolescents for Childcare, Food Production, Domestic Work, Play, and Other Activities (reference) as a function of Net Primary Productivity (NPP). 38

Figure S12. Model 3.6 predictions refitting Model 3 excluding adolescents for Childcare, Food Production, Domestic Work, Play, and Other Activities (reference) as a function of Annual Mean Temperature. 39

5. Site-specific acknowledgements..40
6. References..42

1. Site-specific ethnographic background and detailed data collection methods

1.1. Agta

Agta is a self-describing term used by a population of some 10,000 individuals in the Northern Sierra Madre rainforest on the Philippine island of Luzon[1]. Data for this study were collected by Renée V. Hagen among a community of Agta living on the delta of the river Malibu, in Cagayan Province. Individuals in this community speak the Eastern Cagayan Agta language, and adults are fluent in Ilocano and Tagalog, which are used in communication with outsiders. The Agta are seasonally nomadic, moving camps along rivers and the coastline, settling for longer periods during the monsoon, and staying near towns when agricultural labour is in demand. While some have adopted Christianity, beliefs regarding nature and ancestor spirits are common[2]. Agta in Malibu primarily subsist on hunting of wild pigs, deer, and smaller game, marine and riverine fishing, gathering of wild plants and honey, and cultivated foods (such as rice, vegetables, and luxury items such as coffee, tobacco, sugar, and cookies) bartered with or bought from farming populations. Food is also bought at a local market some five hours' boat ride away. Labour is loosely divided along gender lines. Hunting using bow and arrow or fire weapons is primarily done by men, while gathering is a more female-typical task, although men sometimes gather plant foods and in the current study area some women hunt[3–5]. Men, women, and children fish with spears, although marine fishing is mostly a male endeavor. Mothers provide the bulk of childcare, although alloparents include fathers, siblings, and grandparents, as well as related and unrelated juveniles in children's playgroups[6]. Ethnographic data suggests that early foraging skills are learnt through peers in such playgroups[7]. No schooling was available in or near the camp where data collection took place. Some children attend and board at an elementary school in the closest town, although attendance is generally low and inconsistent.

Data for this study were collected in July and August 2017 through camp scans every two hours between 6:00 and 18:00. During this time the activities, location, and child minder of six to eight randomly selected children were noted. Foraging and childcare could be coded concurrently. The exact ages of some children were known, and ages of others were estimated by ranking all children from most recently to oldest born. Verbal consent of all camp members, including all parents and children over the age of twelve was obtained before commencing the study.

1.2. Aka

The Aka are a northern group of the larger BaYaka population and live in the tropical forests of southwestern Central African Republic (CAR) and northern Congo-Brazzaville. The Aka speak Yaka (or di.Aka), a language derived from the indigenous language of the Ngandu farmers who moved into the region several centuries ago. Many people also speak Sango, the national language of CAR. Aka identify as the "people of the forest", in opposition to the neighbouring farming populations, or "people of the village." Aka society is acephalous and highly egalitarian, and respect for individual autonomy is a core value. Aka live in small communities of as many as 15 nuclear families, though usually only 4 or 5 (around 20-35 people), which fuse and fission throughout the year. Most economic practices are collaborative, and daily life consists of attending to subsistence and maintenance work, often in a leisurely and sociable manner. An average of one-third to one-half of the Aka diet is from cultivated sources, and the rest from hunted game and foraged foods, especially tubers, leaves, nuts, honey, mushrooms, and caterpillars[8,9]. The Aka have gardens, but these are infrequently maintained. Sharing is widespread and often formalized between families in camp[10]. Wage labour and the sale of forest goods for cash to use in village markets was increasing at the time

data were collected, though still relatively minimal, with most daily needs met through subsistence activities or labour exchange. There are four mission schools in Bagandou that Aka children can attend if a child's family or village patron will pay for tuition and supplies. Few Aka attend these schools, and schools were infrequently in session. No schools were available to children inhabiting forest camps.

The data used in this study came from field work conducted by Adam H. Boyette between March and September 2010 with eight Aka residential groups living in association with Bagandou village, Lobaye Province, CAR. Specifically, one group lived on the periphery of the village center, and the other seven lived in forest camps with two- to four-hours walk from the village. Data were recorded using focal follows[11] of individual Aka children, aged four to sixteen, across a random sample of all daylight hours. Focal follows involved following a single individual for a specified period and recording behaviours during predetermined intervals according to a behavioural coding scheme. Children were chosen at random and parental consent and child assent were obtained before beginning observations. Participating children were randomly assigned three two-hour sampling blocks between 6:00 and 18:00 across a series of days. Focal child behaviour was coded each minute using a 30-second-observe, 30-second record procedure. Child age was estimated by Boyette and an Ngandu field assistant, who had known many of the families in the study since childhood. When their individual estimates disagreed by more than two years, parents were asked to place their children in their birth order. By comparing the child in question to ones of known age, an estimated age was derived. Further details and analyses of these data can be found in Boyette[12,13], Boyette and Hewlett[14], and Lew-Levy and Boyette[15]. The Institutional Review Board of Washington State University approved the data collection methods, and permission to conduct the research was given by the Ministre de l'Education Nationale, de l'Ensegnement Supérieur et de la Recherche, Bangui, CAR.

1.3. Baka

The Baka are forest foragers inhabiting the eastern region of Cameroon who share close genetic and cultural ties with the Aka and BaYaka included in the present study. In the 1960s, a government-imposed sedentarization program forced the Baka out of the forest and into settlements alongside roads. Nonetheless, the Baka often forage for forest products for subsistence and for sale. Baka children engage in a variety of subsistence activities such as collective rat hunting, collective bail fishing, butchering, trap-making, and collection of forest products like nuts, caterpillars, and mushrooms[16]. Through these activities, children learn ecological and cultural knowledge[17,18]. The Baka surveyed as part of this research live near Nzime farmers, and these two societies have developed symbiotic relationships over the years. However, some Baka complain about low wages for working in the fields belonging to the Nzime. Baka is the language spoken by Baka children, with Nzime and French words often mixed in. Both public and private primary schools are available at the field site. However, Baka children's attendance at these schools is sporadic because of financial problems, difficulties in balancing work and school, and because of their psychological distance from children in farmer societies[19].

Data for the present study were collected by Koji Sonoda as part of a longitudinal observation of Baka children's participation in everyday activities and chores in villages and forest camps, with most follows conducted in forest camps during the school holidays. The author followed a single child between 6:00 and 18:00 over a single day, continuously noting their behaviours and activities. Play and domestic work activities, and play and food production could be coded concurrently. Two children were followed in a village setting in July and August 2011, and twelve children were sampled in forest camps between September 2012 and August 2013. To estimate age, the author asked each family to rank siblings and cousins by

birth order. Age was then estimated with the help of a fellow researcher who worked in the same population. All research was done with research permits issued by the Ministry of Scientific Research and Innovation of the Republic of Cameroon. All study participants provided informed consent.

1.4. BaYaka

The BaYaka surveyed here, most closely related to the Mbendjele BaYaka[20], live in the dense tropical rainforest of the Congo Basin. Speaking primarily Yaka (di.Aka), many also speak the language of their settled farmer neighbours, with whom they maintain extensive trade relationships[21], and Lingala, the primary trade language used in the northern region of Congo-Brazzaville. BaYaka live in camps of, on average, 22 inhabitants[22]. They participate in a variety of foraging activities, including fishing, hunting with spears and nets, trapping, collecting fruit, greens, mushrooms, nuts, seeds, and caterpillars, and horticultural gardening primarily of bananas, cassava, and other domesticated plants[9,23]. BaYaka maintain egalitarian social relations and have no inherited positions of prestige[20,22]. Food is shared widely[10]. BaYaka are also highly autonomous, and children are rarely reprimanded for refusing to participate in work activities[24]. While BaYaka are increasingly settling into villages, they still spend between three and six months of the year in forest camps, primarily during the *kombi* fishing season (October-December). While free primary education is available in many BaYaka villages, attendance is sporadic, and schools are often closed during heavy rains and when staff payment is delayed. Still, most children spend a few months a year in school. No schooling was available in forest camps, where data for the present study were collected.

Sheina Lew-Levy and Paul Mekouno, a BaYaka field assistant, collected focal follow data among BaYaka in August through September 2017 and 2018, during bail fishing and caterpillar seasons, in six camps in the Likouala department of Congo-Brazzaville. Each child's behaviour was recorded using a 30-second-observe/30-second-record procedure over 2 two-hour sampling blocks scheduled on a single day. Up to two activities could be coded simultaneously. Follows were usually conducted between 8:00 and 10:00, and 12:00 and 14:00, though observation periods were sometimes delayed or paused because of inclement weather or unforeseen social situations (e.g., sudden death of someone in the village). Children were followed both within and outside of camp. To estimate age, children were ranked from oldest to youngest by asking parents about children's birth order, either within a nuclear family, or within a set of closely related cousins, allowing for ties in age. A numerical age was then assigned based on this rank, supplemented with developmental and dental cues. More detailed methods regarding data collection can be found in Lew-Levy et al.[25–27]. Camp-wide consent as well as parent and child verbal consent were obtained before data collection began according to procedures approved by the Cambridge Psychology Research Ethics Committee (PRE.2016.026; PRE.2018.023) and from the Centre de Recherche et D'Etudes en Sciences Sociales et Humaines (CRESSH) and the Institute de Recherche en Sciences Exactes et Naturelles (IRSEN).

1.5. Dukha

The Dukha are nomadic reindeer herders and foragers who occupy portions of the taiga in the Sayan Mountains in Khövsgöl Aimag of northern Mongolia. In all, they number about 200 individuals who are ethnically Tuvan, and though traditionally they spoke the Tuvan language, today everyone speaks the local Darkhad dialect of the Mongolian language. The Dukha are the smallest ethnic minority in Mongolia and likely the southernmost reindeer herders in the world. Dukha subsistence is primarily based on herding of reindeer. Reindeer meat, fat, and milk are dietary staples. Some Dukha families manage herds of other livestock including horses, sheep, goats, cattle, and yak. Hunting and fishing were traditionally part of the

subsistence regime until both were banned by the government in 2011. Gathering of berries, tubers, and pine nuts is common in the late summer and fall. White flour, sugar, salt, and other goods are frequently purchased in town. Tourists regularly visit the taiga in the summer which provides a meagre income stream to Dukha families through sales of handicrafts. Young adult women often leave home to work in the cash economy in the winter. Snow can fall in the Mongolian taiga in any month. In the summer, temperatures occasionally exceed 20°C in the daytime and often drop below freezing at night. Winter lows regularly drop beneath -40°C, and highs are typically around -20°C. Women tend to have more education than men, who are pulled out of school to help with herding in their adolescent years. Dukha camps average about three households and 10 occupants. Childcare responsibility falls primarily on female members of a household, but men, older children, and members of other households regularly help when needed. In the fall and spring, most school-aged children attended school in the nearby town of Tsagaannuur; thus, observations during these field seasons were primarily with children under five and over sixteen. Observations conducted in the summer and winter were primarily during school holidays, when school-aged children were in camp with their families.

Data were collected by Todd Surovell, Matt O'Brien, Randy Haas, Spencer Pelton, and Willa Mullen over five field seasons: July and August 2012, September and October 2014, December and January 2015-16, April to June 2016, and August and September 2017. Data were collected in six different camps, one of which was studied in two different years (2014 and 2017). The primary focus of the research was mapping the distribution of behaviour in Dukha camps. Through that practice, we obtained time allocation for all individuals within Dukha camps. For exterior spaces, we used a time-lapse photographic system to record all activity occurring in daylight hours at 2- to 3-minute intervals. From that imagery, we were able to record all visible individuals and activities. In interior spaces, for 20-minute intervals, in randomly chosen households and times of days, each minute we recorded the activity of a single person, cycling through all individuals in a household. To estimate activities that occurred away from camps, for each house at each camp, we estimated the number of missing observations for each child by comparing the difference between the number of observations of each child and the number of observations of the person with the greatest number of observations. For example, if John was observed at House 1 Camp A 100 times, but Bob was only observed 80 times, Bob has 20 missing observations. To apportion the missing observations into the five behavioural categories, we produced estimates based on additional knowledge of having been present when these data were collected. For example, if a young man spent a large amount of time way from camp in reindeer herding activities, a large percentage of his missing observations can be attributed to food production tasks. Only individuals for whom in-camp data were available and out-of-camp time allocation could be estimated were included in the present analysis. All procedures were approved by the University of Wyoming Institutional Review Board.

1.6. Hadza

The Hadza are a population of equatorial foragers residing in the Lake Eyasi basin of Northern Tanzania. Out of a total of 1,000 individuals who self-identify as Hadza, only approximately 150 people continue to hunt and gather, consuming a mixed subsistence diet that includes small amounts of domesticated foods (e.g., maize, barley), but is primarily composed of gathered plant foods, honey, and wild game. The individuals under study here were practicing a foraging lifestyle at the time of data collection, living in small semi-nomadic camps with fluid residency and composition. Residence patterns were bilocal and flexible, with a slight bias towards living with the wife's family. The Hadza practice bilateral descent, do not recognize clans, and are largely serially monogamous, although a small number of polygynous relationships has been reported to anthropologists over the past sixty years. There is a strong gendered division of

labour, with women targeting plant foods and men targeting honey and animal products, including avian, riparian, and game meat. Infants forage with their mothers until they are weaned and able to walk considerable distances on their own—at which point they will join in to large mixed-gender mixed-age play groups and foraging parties[25]. At the time of data collection, children were routinely contributing to the foraging economy, able to successfully collect a large portion of their daily calories by middle childhood[28]. While two schools were within travel distance to the study site by car, no participating children were attending or had ever attended school[29].

Alyssa Crittenden collected data from January to October 2005 in two camps during a long-term project on children's foraging, play, and social learning. Focal follow data were collected during daylight hours for three-hour time blocks. Activities were recorded at five-minute intervals. Age was derived from three waves of census data starting in 1982[28]. Camp-wide research consent, parental consent, and child assent were all obtained verbally before data collection began. All procedures were approved by the University of California, San Diego Human Research Subjects Institutional Review Board (PI Alyssa Crittenden) and the Harvard University Committee on the Use of Human Subjects (PI Frank Marlowe). All research was done with the permission of the Tanzanian Commission for Science and Technology (COSTECH).

1.7. Matsigenka

At the time of data collection, the Matsigenka, a population of Arawakan-speakers, numbered approximately 10,000, with the majority living in the dense tropical forest along the Urubamba river in Southeastern Peru[30]. The Matsigenka were primarily slash and burn agriculturalists of cassava and corn, and also hunted small birds and mammals, fished, collected wild fruit and palm hearts[30], and kept chickens and ducks[31]. Men primarily undertook garden labour and fishing. Hunting was exclusively conducted by men. Both men and women participated in foraging. Historically semi-nomadic, at the time of data collection, the Matsigenka were increasingly living in larger settlements of several hundred individuals organized into extended-family compounds[31,32]. Matsigenka were relatively egalitarian, with no institutionalized leadership. Marriage was primarily exogenous and monogamous, with polygyny occurring in approximately 30% of households. Residence was primarily matrilocal and flexible[30]. Communities were relatively remote, and only accessible by boat or foot. Market integration was limited, with community members visiting nearby market towns 2-6 times a year[31]. Younger children played in multi-aged and mixed-gender groups of siblings and cousins, while older children segregated by gender to forage and explore. Children were socialized for self-reliance from an early age[33]. At the time of data collection, elementary schools were available to children.

Data for the Matsigenka comes from the Human Relation Area Files Cross-Cultural Studies in Time Allocation. Data from Shimaa was collected by Johnson and Johnson in August 1972 through July 1973[31] and from Camana by Michael Baksh in September 1979 through October 1980[32]. In both cases, the dataset is made up of spot observations[34]. Households were sampled randomly on randomly allocated days during randomly allocated daylight hours, with most households being observed once per day. We included only individuals that had been observed 15 or more times to ensure appropriate coverage. Estimates for proportion of farmed and foraged foods, and the Gendered division of labour associated with these foods, were calculated using values published in Johnson[33].

1.8. Maya

The Maya are subsistence maize farmers who live in the low canopy tropical forests of the Puuc region in the interior of the Yucatan Peninsula, Mexico. At the time of data collection,

villagers cultivated maize, beans, and squash, depended on some domestic animals, hunted game, and collected forest plants. Honey and small quantities of maize might be exchanged for limited goods. Otherwise, no cash crops were grown. Families lived in wattle-and-daub dirt-floor houses. There was no electricity or running water and limited access to motorized transportation. Each household grew its own food and furnished the labour to provision the household. Maya children lived and worked in their parents' households until they married and began families of their own in their late teens to early twenties. As soon as children can walk, they frequently are found away from their homes visiting friends and relatives, running errands, working in the fields, or accompanying their parent into the forest. By the age of six to eight, Maya children participate in a variety of domestic and field activities. The increase in the time spent working then sharply rises at about age 10 and reaches a plateau, approaching adult levels of work in their mid-teens. Maya is a child’s first and primary language. Despite their predominantly maize diet, Maya children were well-fed and in generally good health. Child mortality was low; 96% of children survive to reproductive age. Child mortality was probably low aboriginally, in part because water is drawn from closed limestone wells; there are no rivers or flowing water in this region of the Yucatan, attenuating one of the primary vectors of child mortality. While there was a rustic primary school, classes were infrequently held and school rarely interrupted children's work activities. Some children also attended boarding school.

The time allocation data for this study were collected by Karen Kramer[35] from April 1992 to March 1993 as part of a longitudinal demographic and life history project. Instantaneous scan samples were collected over the course of a year. An observation period lasted three to four hours, during which a participant's activity was recorded every 15 minutes. The observation day was limited by daylight hours from 7:00 to 18:00. Variables recorded during a scan sample included the individual, his or her activity, the object of the activity, location, date, and time. Children’s behaviour was coded using a hierarchical coding scheme that included over 400 possible activities. Activities could be coded concurrently. The sample included about 40% of village children. Ages are known to the month and year. Participant verbal consent was obtained prior to commencing the study.

1.9. Mayangna

The Mayangna are indigenous horticulturalists who reside in the forested areas of northeastern Nicaragua. They primarily live in sedentary communities along the rivers of the region. In terms of subsistence, the Mayangna complement their horticultural activities with fishing, hunting, and the consumption of small livestock. For monetary income, artisanal gold panning is the primary source of income for many households, though a smaller number of individuals earn money via mercantilism and government contracts as schoolteachers. Men are responsible for most agricultural activities and hunting. For other activities, including fishing and gold panning, women sometimes accompany their husbands and other male relatives to provide assistance[36]. Wealth inequality is high. There are no permanent political positions. The Mayangna exhibit an uxorilocal residence pattern. Descent is traced bilaterally. At the time of data collection, fertility was high, with total fertility rates exceeding 7 births per woman. Before the age of 13, children rarely leave the community unaccompanied by older individuals. Adolescents occasionally embark on solitary excursions, such as gathering firewood or fishing, but in general, they are also likely to accompany older individuals. School is normatively compulsory for children between ages 5 and 12. At the time of data collection, participation in schooling by adolescents was sporadic and largely occurring via boarding schools outside of the study community.

Jeremy Koster collected instantaneous scan sampling data among the Mayangna from September 2004 to August 2005 in a community of approximately 25 households.

Observations were scheduled during daylight hours. The initial observation was scheduled randomly between 5:30 and 6:00, and then subsequent observations were scheduled every 30 minutes, concluding no later than 18:00. Observations were organized by household. During an observation, the lead author documented the activities of all household residents. Households were sampled without replacement such that no household was observed more than once per day. Ages for children were based on a combination of government records, reproductive histories, and comparative methods of anthropological demography. Consent was obtained at a community meeting at the outset of the project, and protocols were approved by the Institutional Review Board at Penn State University.

1.10. Mikea

Mikea live in and around the dry, deciduous forests of Madagascar's semi-arid southwest. Mikea are the descendants of Masikoro agropastoralists and Vezo fishers who, within the past four centuries, adopted a forest-based life and foraging subsistence to escape domination by the precolonial kings, and later, the French colonial authority. Whereas the recently created Mikea Forest National Park claims that there are around 1,000 Mikea people, this does not count the many thousands of self-described Mikea who live in large, permanent villages and who move into and out of forest life depending on their socioeconomic circumstances. The data presented here were collected in a forest camp where Mikea gathered wild tubers and honey and hunted small animal prey while also participating in a market boom for maize grown in swidden fields. Although hunting and gathering is central to Mikea identity, Mikea have probably always combined foraging with some cattle and goat herding, and cultivation of manioc, maize, and other crops. Mikea routinely interact with neighbouring agropastoral Masikoro and coastal Vezo, through market exchange, intermarriage, and ceremonies[37]. Mikea belong to Masikoro or Vezo patrilineal clans. Like all Malagasy, they revere ancestors whom they invoke through ritual livestock sacrifice. No schools were available to children living in the camp where data collection took place.

Bram Tucker, working with colleagues Tsiazonera, Jaovola Tombo, and Tsimitamby from the Université de Toliara, Madagascar, collected the Mikea data presented here during Tucker's dissertation research at the forest camp of Belò over nine non-consecutive months spanning all four of the locally defined seasons between 1996-1999 before the creation of the Mikea Forest National Park. To randomly schedule each day's scan sampling observations, at the beginning of each sampling month, the researchers wrote on to bits of paper the daylight hours at half-hour increments, and then drew the papers randomly from a hat. The result was two or three random observations per day. At each observation, we recorded the location and activity of all camp members and visitors; those not in view were coded as "away" along with the purpose of their absence (foraging, farming, etc.). To classify ages, researchers ranked participants by age, and then defined categories based on informants' classifications. Here, we included only permanent residents, and participants aged as early juvenile (5-8 years), late juvenile (9-15 years), and young adults (16-25 years). We obtained community consent during public fora and the consent of individuals before interviews. All methods were approved by the Institutional Review Board of the University of North Carolina at Chapel Hill.

1.11. Savanna Pumé

The Savanna Pumé are mobile hunter-gatherers indigenous to the *llanos* (savannas) of west-central Venezuela. The Savanna Pumé number about 650, dispersed in 24 bands over a 2,800 square kilometer area. Because of the region's political instability, their geographic isolation, and a poor terrestrial environment, the Savanna Pumé are largely buffered from outside encroachment and continue to live a hunting and gathering way of life. The Savanna Pumé are related to the River Pumé, their horticultural neighbours, with whom they exchange some

goods, and infrequently, marriage partners. The Savanna Pumé move 5 to 6 times a year in response to changes in rainfall and the water table. During the six-month dry season, food is relatively abundant and subsistence centers on aquatic resources and wild fruit. When the *llanos* flood during the wet season, fish are difficult to locate and the subsistence base shifts to terrestrial game and tubers. Males, both adults and children, almost exclusively fish and hunt, while women and girls gather tubers and do most of the food processing. Both men and women collect wild fruit. Male- and female-foraged foods are both critical to the diet and are widely shared within and across families. Women and children provide most of the childcare. They are monolingual, and Pumé is a child's first and only language. Savanna Pumé women marry on average at age 15.1 (SD ±2.5; n=59) and males at age 18.0 (SD ±4.3; n=51). Although first marriages are often arranged, young people are not obliged to accept these matches, and have autonomy about when and whom they marry. The Savanna Pumé have no schools, and no access to health care or market foods.

The data for this study were collected in April to July 2006 and 2007 by Karen Kramer and Russell D. Greaves[38] as part of a longitudinal demographic and life history project. The time allocation data were collected using instantaneous scan sampling in one band of Savanna Pumé, who numbered 73 adults and children at the time of the study. All children were included in the scan sampling. Daylight hours (from 7:00 to 18:30) were broken into three blocks, two of which were sampled during each data collection day. During a block, a scan sample was recorded once an hour for everyone in camp. Variables recorded during a scan sample included the individual, his or her activity, the object of the activity, location, date, and time. Children's behaviour was coded using a hierarchical coding scheme that included over 400 possible activities. Although the Savanna Pumé do not use ordinal numbers greater than ten, ages are known to the year using several ethnographic methods including recording reproductive histories from multiple relatives and in kin terms, which are specific to gender, birth order and rank. Many children's ages can also be anchored to observed births during or shortly before field seasons. In the present analysis, we excluded children who were in our age range but who were parents at the time of data collection. Participant verbal consent was obtained prior to commencing the study. Research protocols were approved by the Committee for the Protection of Human Subjects at Stony Brook University and Harvard University.

1.12. Tsimane

The Tsimane are semi-sedentary forager–horticulturalists of the Bolivian Amazon, inhabiting more than 90 villages which range in size from 50 to 500 individuals. Villages are composed of household clusters, each of which typically contains three or four residences of consanguineal or affinal kin. They cultivate plantains, rice, corn, and sweet manioc in small swiddens, and regularly fish and hunt game. These foods together provide more than 90% of the calories in their diet, with the remainder coming mainly from store-bought items or trade with itinerant merchants. Most marriages are monogamous. Women's mean age at first marriage is 17 years, and their mean age at first birth is 18 years[39]. There are no rules of postmarital residence, but the norm is residence near the wife's natal kin early in marriage. Spouses engage in extensive cooperation and gender roles are well-defined. Women are responsible for providing childcare and preparing food and *chicha* (homemade beer). Men acquire game and fish and engage in wage labour. Both genders collect forest fruits, fetch firewood and water, and work in horticultural gardens. Schools exist in most villages, although attendance is sporadic.

Scan sampling was conducted in four communities between June 2002 and June 2003, and in an additional three communities throughout 2005 and 2006 by graduate students on the Tsimane Health and Life History Project[40], including Jonathan Stieglitz and Helen E. Davis. To collect time allocation data, households were first divided into residential clusters, typically

consisting of extended families. Each cluster was sampled randomly without replacement from 7:00 to 19:00 in three-hour time blocks in 2002-2003, and in two-hour time blocks in 2005. During these time blocks, the activity, location, and interactants were recorded every half-hour. An individual could be coded as participating in up to two activities simultaneously. For this study, only individuals who had more than 50 instantaneous scans were included in analyses. If coded activities were not directly observed by the researcher or later confirmed by the individual in question, the scan was not included in analyses. Individuals within the sampled age range for the present study who were parents were excluded. Consent was provided from the Tsimane government (Gran Consejo Tsimane), village leadership, and study participants. All procedures were approved by the IRB at the University of New Mexico and the University of California-Santa Barbara.

2. Supplementary Methods, Results, and Discussion

2.1. Correlation of random effects

To examine trade-offs in time allocation to play and work activities, Model 1 included only the random effects of individual and society (Table S10, Figure S1). As in previous time allocation studies, there were strong and negative correlations between the random effects of play and childcare ($\rho = -0.17$), play and food production, ($\rho = -0.22$), and play and domestic work ($\rho = -0.36$), indicating individual-level trade-offs. In contrast, correlated individual random effects for all work activities were strong and positive (childcare and food production; $\rho = 0.20$, childcare and domestic work; $\rho = 0.51$, food production and domestic work; $\rho = 0.44$).

Several studies demonstrate that children's time allocation to play and work trades off with age, and that this trade-off occurs earlier for girls than for boys[12,15,41,42]. Thus, much individual-level trade-off in children's participation in play and work may be explained by age and gender. As a result, the correlations of individual-level random effects were affected by the introduction of age and gender as fixed effects into Model 2 (Table S11; Figure S2), since "random effects are estimated in relation to the effects of all covariates"[43]. In other words, the correlation of individual-level random effects in Model 2 can be interpreted as trade-offs in time allocation when adjusting for participant age and gender. In Model 2, play was positively correlated with childcare ($\rho = 0.22$) and with domestic work ($\rho = 0.15$), potentially because children can easily transition from play to childcare and/or domestic work and back again to distract and/or soothe children while mothers and others are engaged in camp- or village-based work. The correlation between domestic work and food production ($\rho = 0.25$), and domestic work and childcare ($\rho = 0.24$) remained strong and positive, likely because beyond gender and age, participation in these activities increased simultaneously with children's individual skill[44]. The correlations between food production and play ($\rho = 0.08$), and food production and childcare ($\rho = 0.08$) were weakened, suggesting that the strong correlation found in Model 1 was mostly attributable to participant age and gender.

2.2. Additional correlates for cross-cultural variation in time allocation

Coefficient of Variation of Monthly Precipitation (CV %). CV % was calculated using the University of East Anglia Climate Research Unit gridded Time Series dataset[45]. Using the *raster*[46] package in R[47], we extracted total monthly precipitation (mm) for each field site. Monthly values were averaged over the 30 years preceding and including the most recent year of behavioural data collection for each field site. We then used these monthly values to calculate CV % using the biovars function in the *dismo*[48] package. The specific formulas used

for this variable can be found in O'Donnell and Ignizio[49]. CV % was not a strong predictor for participation in work and play (Table S17; Figure S9).

Medically Important Venomous Snakes. Medically Important Venomous Snake Species for each country were extracted from data available in Longbottom et al.[50]. The range map for each species was visually inspected by IP to identify species present at each field site. Species presence/absence was then confirmed by each ethnographer. Using the finalized list, we calculated the total number of Medically Important Venomous Snake species at each site. Total number of Medically Important Venomous Snake species was not a strong predictor for participation in work and play (Table S17; Figure S10).

2.3. Assessment of multicollinearity and verification of model results

Collinearity between independent variables can make regression coefficients difficult to interpret. We investigated collinearity for Models 3-5 and found Variance Inflation Factors (VIF) to be ≤2.67 See Table S7-9 for variable correlations and VIFs.

We verified our model results by estimating versions of Models 3 and 4 that included each environmental factor and ecological risk effect individually. The posterior estimates for these parameters did not change substantially in these new fits, indicating that the coefficient estimates reported for the full models in Table S14 are reliable.

As stated in the limitations of the main text, the small sample of societies hindered our ability to account for potential interaction effects. It is possible that environment and ecological risk predominantly constrain the time allocation of younger children, whereas adolescent activity patterns may more closely resemble those of adults. To test this possibility, we refit Models 3 and 4 excluding adolescents. Table S16 shows that estimates for local ecological risk remain consistent with those reported in the text. For environmental factors, estimates also remain consistent, though the model is more certain about the effect of Net Primary Productivity on play, and Annual Mean Temperature on domestic work. Comparisons of Figures S3 with S11, and Figures S4 with S12, however, show that the effects reported in the text for the entire sample, and those excluding adolescents, are consistent, and that the effect of Annual Mean Temperature on participation in domestic work is relatively flat.

3. Supplementary Tables

Table S1. Description of activities coded as childcare, food production, domestic work, play, and other activities by sampled society.

	**Childcare**	**Food production**	**Domestic work**	**Play**	**Other Activities**
Agta	Carrying, holding, soothing, bathing, feeding, reprimanding, playing with, sitting close to	Spear fishing, net fishing, collecting shellfish, collecting plant foods	Washing clothes, preparing food, cleaning the house, collecting water, washing dishes	Solitary play, group play, playing house, games, swimming, running around	resting, eating, drinking, personal hygiene, crying
Aka	Holding, carrying, soothing, cleaning, bathing, nursing, feeding, playing	Gathering, net hunting, spear hunting, making snares, other hunting, collecting honey, fishing, gardening	Food preparation, house construction, miscellaneous work	Solitary and social bject play, exercise play, rough-and-tumble play, gentle-and-tumble play, structured games, pretense play, roaming play	Visit, music, rest, travel, maintenance, other activities
Baka	Holding, playing with, tending to, feeding, nurturing	Gathering, gardening, collective hunting, setting traps, fishing	Food processing, cooking, housekeeping, fetching water and firewood, doing dishes, laundry	Solitary and social object play soccer, playing house, structured games	Taking a walk, chatting, music, idle object manipulation
BaYaka	Holding, soothing, bathing, feeding, changing, distracting	Collecting caterpillars, liana fruit, greens, gardening, tubers, setting traps, hunting with guns and spears, fishing	Sweeping, doing dishes, laundry, fetching water and firewood, food processing, cooking, fixing and manufacturing subsistence tools	Solitary and social object play, exercise play, rough-and-tumble play, gentle-and-tumble play, structured games, pretense play, roaming play	Travel, rest, music, eating, hygiene
Dukha	Feeding, carrying, distracting, holding, soothing	Foraging, hunting, herding	Tool manufacture and repair, camp cleaning, house construction, firewood, fetching water, cleaning house, cooking, doing dishes, packing camp	Playing house, climbing, wrestling, playing with objects, playing with friends	Hygiene, eating, resting, socializing, music, sleeping, walking around
Hadza	Playing, holding, nurturing, tending to an injury, feeding	Collecting fruit/greens/honey, digging tubers, setting traps, hunting	Food processing (e.g. pounding, winnowing)	Physical/locomotor play, object play, fantasy/social play, structured play, solitary play, exercise play, work play	Eating, walking. resting
Matsigenka	Actively tending to child	Agriculture, wild plant foods, aquatic collecting, hunting, tending food, draft animals	Housekeeping, fetching and managing household water and fuel, other housework, making and repairing artefacts, clothes and facilities, acquiring materials, handling, storing, and cooking	General play, soccer	Eating, hygiene, religious activities, socializing, school
Maya	Feeding, bathing, dressing, grooming, carrying, holding, indirect childcare	Field work, harvesting, weeding, beekeeping, tending animals	Domestic work, collecting firewood, water, cleaning, washing, sewing, cooking, food processing, food preparation	Plays with others, games, sport, plays alone	Resting, visiting, personal maintenance, eating, out-of-village unobserved activity, school
Mayangna	Holding, soothing, bathing, feeding, entertaining, protecting	Horticulture, fishing, hunting, livestock care	Laundering clothes, washing dishes, cooking, cleaning	Swimming, soccer, pretence play, climbing, exercise play	Resting, school, personal care, attending church
Mikea	Childcare	Agricultural work, gathering, hunting, honey collecting, keeping livestock, fishing	Collecting firewood, house building, cleaning, fetching water, food preparation, making and repairing tools	Social recreation, individual recreation, dominos	Socializing, hygiene, eating, resting
Pumé	Nursing, feeding, bathing, dressing, grooming, carrying, holding, indirect childcare	Fruit and root foraging, fishing, hunting	Collecting firewood, collecting water, cooking, food processing, food preparation, cleaning, washing, sewing, weaving, making and repairing tools and other	Plays with others, plays alone	Resting, visiting, personal maintenance, eating, out-of-camp unobserved activity
Tsimane	Bathing, grooming, cooing, feeding, holding, comforting, reprimanding, holding	Fishing, agricultural work, collecting, hunting	Cleaning house, domestic animal care, fetching firewood and water, food processing, house building, tool repair and manufacture	Plays with others, plays with self, house, chase, soccer, manufacture, balance, imitation of adult work	Hygiene, sleeping, talking, school and schoolwork, meetings, walk, eating, attending church

Table S2. Time allocation by society, gender, and age. Binomial 90% confidence intervals are in parentheses. Values represent population proportion of observation time. Early childhood represents children between 3 and 6 years. Middle childhood represents children between 7 and 12 years. Adolescence represents children between 13 and 18 years[1].

Society	Age Category	Gender	Childcare	Food Production	Household	Play
Agta	Early Childhood	Girls	0 (0, 0.04)	0.01 (0, 0.06)	0 (0, 0.04)	0.39 (0.3, 0.5)
Agta	Early Childhood	Boys	0 (0, 0.02)	0.02 (0, 0.05)	0.01 (0, 0.04)	0.44 (0.36, 0.51)
Agta	Middle Childhood	Girls	0.14 (0.07, 0.24)	0.14 (0.07, 0.24)	0.04 (0.01, 0.12)	0.16 (0.08, 0.27)
Agta	Middle Childhood	Boys	0.08 (0.03, 0.16)	0.17 (0.1, 0.27)	0.02 (0, 0.07)	0.25 (0.16, 0.35)
Aka	Early Childhood	Girls	0.01 (0.01, 0.01)	0.05 (0.04, 0.06)	0.04 (0.04, 0.05)	0.31 (0.3, 0.33)
Aka	Early Childhood	Boys	0 (0, 0)	0.02 (0.02, 0.03)	0.04 (0.03, 0.05)	0.48 (0.46, 0.5)
Aka	Middle Childhood	Girls	0.01 (0.01, 0.01)	0.14 (0.13, 0.16)	0.12 (0.11, 0.14)	0.18 (0.17, 0.2)
Aka	Middle Childhood	Boys	0.02 (0.02, 0.03)	0.04 (0.03, 0.05)	0.05 (0.04, 0.06)	0.31 (0.3, 0.33)
Aka	Adolescence	Girls	0.03 (0.02, 0.04)	0.2 (0.18, 0.21)	0.15 (0.14, 0.16)	0.07 (0.06, 0.08)
Aka	Adolescence	Boys	0 (0, 0.01)	0.11 (0.1, 0.12)	0.06 (0.05, 0.07)	0.16 (0.15, 0.18)
Baka	Early Childhood	Girls	0 (0, 0)	0.32 (0.31, 0.34)	0.01 (0.01, 0.02)	0.21 (0.19, 0.22)
Baka	Early Childhood	Boys	0.01 (0.01, 0.02)	0.09 (0.08, 0.11)	0.02 (0.01, 0.02)	0.48 (0.46, 0.5)
Baka	Middle Childhood	Girls	0.05 (0.04, 0.06)	0.03 (0.02, 0.03)	0.17 (0.15, 0.19)	0.15 (0.14, 0.17)
Baka	Middle Childhood	Boys	0 (0, 0)	0.29 (0.28, 0.3)	0.1 (0.09, 0.11)	0.27 (0.26, 0.28)
Baka	Adolescence	Girls	0 (0, 0)	0.44 (0.42, 0.46)	0.18 (0.16, 0.19)	0.08 (0.07, 0.09)
Baka	Adolescence	Boys	0 (0, 0)	0.37 (0.34, 0.4)	0.11 (0.09, 0.13)	0.1 (0.09, 0.12)
BaYaka	Early Childhood	Girls	0.01 (0, 0.01)	0.01 (0.01, 0.02)	0.06 (0.05, 0.08)	0.41 (0.39, 0.44)
BaYaka	Early Childhood	Boys	0.01 (0, 0.01)	0.03 (0.02, 0.04)	0.02 (0.02, 0.03)	0.33 (0.31, 0.36)
BaYaka	Middle Childhood	Girls	0.03 (0.03, 0.04)	0.08 (0.07, 0.09)	0.18 (0.17, 0.2)	0.26 (0.25, 0.28)
BaYaka	Middle Childhood	Boys	0.01 (0.01, 0.01)	0.05 (0.05, 0.06)	0.04 (0.04, 0.05)	0.34 (0.33, 0.36)
BaYaka	Adolescence	Girls	0.06 (0.05, 0.07)	0.33 (0.31, 0.35)	0.14 (0.13, 0.15)	0.06 (0.05, 0.06)
BaYaka	Adolescence	Boys	0.01 (0.01, 0.01)	0.19 (0.18, 0.2)	0.1 (0.09, 0.11)	0.26 (0.24, 0.27)
Dukha	Early Childhood	Girls	0 (0, 0)	0 (0, 0.01)	0.03 (0.02, 0.05)	0.08 (0.06, 0.1)
Dukha	Early Childhood	Boys	0 (0, 0)	0.01 (0, 0.01)	0.01 (0.01, 0.02)	0.23 (0.22, 0.25)
Dukha	Middle Childhood	Girls	0.01 (0.01, 0.02)	0.01 (0.01, 0.02)	0.11 (0.09, 0.13)	0.08 (0.07, 0.1)
Dukha	Middle Childhood	Boys	0 (0, 0.01)	0 (0, 0.01)	0.02 (0.01, 0.05)	0.18 (0.14, 0.23)
Dukha	Adolescence	Girls	0 (0, 0.01)	0.11 (0.09, 0.13)	0.18 (0.15, 0.2)	0.01 (0, 0.02)
Dukha	Adolescence	Boys	0.01 (0.01, 0.01)	0.29 (0.28, 0.3)	0.13 (0.12, 0.14)	0.03 (0.03, 0.04)
Hadza	Early Childhood	Girls	0.05 (0.02, 0.09)	0.22 (0.16, 0.28)	0.04 (0.02, 0.08)	0.29 (0.22, 0.36)
Hadza	Middle Childhood	Girls	0.02 (0.01, 0.04)	0.25 (0.21, 0.31)	0.16 (0.12, 0.21)	0.2 (0.15, 0.25)
Hadza	Middle Childhood	Boys	0 (0, 0.02)	0.24 (0.19, 0.3)	0.02 (0, 0.04)	0.03 (0.01, 0.06)
Hadza	Adolescence	Girls	0 (0, 0.21)	0.31 (0.11, 0.57)	0 (0, 0.21)	0 (0, 0.21)
Hadza	Adolescence	Boys	0.03 (0.01, 0.08)	0.1 (0.06, 0.17)	0 (0, 0.03)	0.04 (0.01, 0.09)
Matsigenka	Early Childhood	Girls	0 (0, 0.01)	0.02 (0.01, 0.03)	0.02 (0.01, 0.03)	0.09 (0.08, 0.11)
Matsigenka	Early Childhood	Boys	0 (0, 0.01)	0.03 (0.02, 0.05)	0.01 (0, 0.03)	0.09 (0.06, 0.12)
Matsigenka	Middle Childhood	Girls	0.04 (0.03, 0.05)	0.06 (0.04, 0.07)	0.09 (0.07, 0.11)	0.08 (0.06, 0.1)
Matsigenka	Middle Childhood	Boys	0.01 (0, 0.01)	0.17 (0.14, 0.2)	0.08 (0.06, 0.1)	0.2 (0.17, 0.23)
Matsigenka	Adolescence	Girls	0.06 (0.04, 0.08)	0.13 (0.11, 0.16)	0.24 (0.21, 0.27)	0.01 (0.01, 0.03)
Matsigenka	Adolescence	Boys	0 (0, 0.02)	0.26 (0.21, 0.31)	0.12 (0.09, 0.16)	0.09 (0.06, 0.12)
Maya	Early Childhood	Girls	0.05 (0.04, 0.06)	0.03 (0.03, 0.04)	0.06 (0.05, 0.08)	0.45 (0.42, 0.47)
Maya	Early Childhood	Boys	0.01 (0.01, 0.02)	0.02 (0.01, 0.03)	0.03 (0.02, 0.04)	0.7 (0.68, 0.72)
Maya	Middle Childhood	Girls	0.12 (0.11, 0.14)	0.05 (0.04, 0.06)	0.26 (0.24, 0.28)	0.19 (0.17, 0.21)
Maya	Middle Childhood	Boys	0.04 (0.03, 0.05)	0.14 (0.12, 0.16)	0.12 (0.1, 0.14)	0.27 (0.24, 0.29)
Maya	Adolescence	Girls	0.06 (0.05, 0.07)	0.1 (0.09, 0.11)	0.5 (0.48, 0.52)	0 (0, 0.01)
Maya	Adolescence	Boys	0.01 (0, 0.02)	0.19 (0.16, 0.22)	0.11 (0.09, 0.14)	0.02 (0.01, 0.03)
Mayangna	Early Childhood	Girls	0.01 (0.01, 0.02)	0 (0, 0)	0.04 (0.03, 0.05)	0.32 (0.3, 0.35)
Mayangna	Early Childhood	Boys	0 (0, 0)	0 (0, 0.01)	0.02 (0.02, 0.03)	0.43 (0.41, 0.45)
Mayangna	Middle Childhood	Girls	0.07 (0.05, 0.08)	0.04 (0.03, 0.05)	0.26 (0.24, 0.28)	0.11 (0.09, 0.12)
Mayangna	Middle Childhood	Boys	0.01 (0.01, 0.02)	0.07 (0.06, 0.09)	0.12 (0.11, 0.14)	0.19 (0.18, 0.21)
Mayangna	Adolescence	Girls	0.04 (0.03, 0.05)	0.07 (0.06, 0.08)	0.44 (0.42, 0.47)	0.01 (0.01, 0.02)
Mayangna	Adolescence	Boys	0 (0, 0)	0.18 (0.16, 0.2)	0.19 (0.17, 0.21)	0.07 (0.06, 0.09)
Mikea	Early Childhood	Girls	0 (0, 0)	0.09 (0.07, 0.11)	0.03 (0.02, 0.04)	0.35 (0.32, 0.38)
Mikea	Early Childhood	Boys	0 (0, 0.01)	0.24 (0.21, 0.28)	0.02 (0.01, 0.04)	0.3 (0.27, 0.34)
Mikea	Middle Childhood	Girls	0.01 (0, 0.01)	0.21 (0.19, 0.23)	0.1 (0.09, 0.11)	0.22 (0.2, 0.24)
Mikea	Middle Childhood	Boys	0 (0, 0.01)	0.31 (0.29, 0.33)	0.07 (0.06, 0.08)	0.2 (0.18, 0.22)
Mikea	Adolescence	Girls	0 (0, 0.02)	0.23 (0.19, 0.28)	0.24 (0.2, 0.29)	0.03 (0.02, 0.06)
Mikea	Adolescence	Boys	0 (0, 0.01)	0.46 (0.42, 0.49)	0.04 (0.03, 0.06)	0.07 (0.05, 0.09)
Pumé	Early Childhood	Girls	0.01 (0, 0.03)	0 (0, 0.02)	0.05 (0.02, 0.09)	0.17 (0.12, 0.22)
Pumé	Early Childhood	Boys	0.01 (0, 0.01)	0.09 (0.07, 0.1)	0.05 (0.04, 0.06)	0.42 (0.39, 0.44)
Pumé	Middle Childhood	Girls	0.08 (0.07, 0.1)	0.05 (0.04, 0.07)	0.17 (0.14, 0.19)	0.12 (0.1, 0.14)
Pumé	Middle Childhood	Boys	0.02 (0.01, 0.03)	0.25 (0.23, 0.27)	0.11 (0.09, 0.12)	0.18 (0.16, 0.2)
Pumé	Adolescence	Girls	0.05 (0.05, 0.06)	0.08 (0.07, 0.09)	0.34 (0.32, 0.36)	0.02 (0.01, 0.02)
Pumé	Adolescence	Boys	0.02 (0.01, 0.03)	0.24 (0.21, 0.28)	0.13 (0.1, 0.15)	0.06 (0.04, 0.08)

Tsimane	Early Childhood	Girls	0.02 (0.02, 0.03)	0.02 (0.02, 0.03)	0.07 (0.06, 0.08)	0.23 (0.21, 0.24)
Tsimane	Early Childhood	Boys	0.01 (0.01, 0.01)	0.02 (0.01, 0.02)	0.08 (0.07, 0.08)	0.3 (0.28, 0.31)
Tsimane	Middle Childhood	Girls	0.06 (0.05, 0.07)	0.05 (0.04, 0.06)	0.18 (0.17, 0.19)	0.12 (0.1, 0.13)
Tsimane	Middle Childhood	Boys	0.01 (0, 0.01)	0.07 (0.06, 0.08)	0.1 (0.09, 0.11)	0.28 (0.26, 0.29)
Tsimane	Adolescence	Girls	0.09 (0.07, 0.1)	0.08 (0.07, 0.1)	0.31 (0.29, 0.33)	0.03 (0.03, 0.04)
Tsimane	Adolescence	Boys	0.01 (0, 0.01)	0.09 (0.08, 0.11)	0.16 (0.14, 0.18)	0.08 (0.07, 0.1)
**Total**	**Early Childhood**	**Girls**	**0.01 (0.01, 0.02)**	**0.08 (0.08, 0.09)**	**0.04 (0.04, 0.05)**	**0.28 (0.27, 0.28)**
**Total**	**Early Childhood**	**Boys**	**0.01 (0, 0.01)**	**0.04 (0.03, 0.04)**	**0.03 (0.03, 0.04)**	**0.39 (0.38, 0.4)**
**Total**	**Middle Childhood**	**Girls**	**0.05 (0.04, 0.05)**	**0.08 (0.08, 0.08)**	**0.17 (0.16, 0.17)**	**0.17 (0.16, 0.17)**
**Total**	**Middle Childhood**	**Boys**	**0.01 (0.01, 0.01)**	**0.14 (0.13, 0.14)**	**0.08 (0.08, 0.08)**	**0.27 (0.26, 0.27)**
**Total**	**Adolescence**	**Girls**	**0.04 (0.04, 0.05)**	**0.18 (0.18, 0.19)**	**0.28 (0.27, 0.28)**	**0.04 (0.03, 0.04)**
**Total**	**Adolescence**	**Boys**	**0.01 (0.01, 0.01)**	**0.23 (0.22, 0.23)**	**0.11 (0.11, 0.12)**	**0.12 0.11, 0.12)**

[1]Exact ages for Mikea children were not known. Children were instead categorized as early juveniles (5-8 years), late juveniles (9-15 years), and young adults (16-25 years), corresponding to early childhood, middle childhood, and adolescence, respectively, in the present table.

Table S3. Time allocation by society and age. Binomial 90% confidence intervals are in parentheses. Values represent population proportions of observation time. Early childhood represents children between 3 and 6 years. Middle childhood represents children between 7 and 12 years. Adolescence represents children between 13 and 18 years[1].

Society	Age Category	Childcare	Food Production	Domestic Work	Play
Agta	Early Childhood	0 (0, 0.01)	0.01 (0, 0.04)	0 (0, 0.02)	0.42 (0.36, 0.48)
Agta	Middle Childhood	0.1 (0.06, 0.16)	0.16 (0.1, 0.22)	0.03 (0.01, 0.07)	0.21 (0.15, 0.28)
Aka	Early Childhood	0.01 (0, 0.01)	0.04 (0.03, 0.04)	0.04 (0.04, 0.05)	0.39 (0.38, 0.4)
Aka	Middle Childhood	0.02 (0.01, 0.02)	0.09 (0.08, 0.1)	0.09 (0.08, 0.09)	0.25 (0.24, 0.26)
Aka	Adolescence	0.02 (0.01, 0.02)	0.16 (0.15, 0.17)	0.11 (0.1, 0.12)	0.11 (0.1, 0.12)
Baka	Early Childhood	0.01 (0, 0.01)	0.23 (0.22, 0.24)	0.01 (0.01, 0.02)	0.32 (0.31, 0.33)
Baka	Middle Childhood	0.02 (0.01, 0.02)	0.2 (0.19, 0.21)	0.13 (0.12, 0.13)	0.23 (0.22, 0.24)
Baka	Adolescence	0 (0, 0)	0.42 (0.4, 0.44)	0.15 (0.14, 0.17)	0.09 (0.08, 0.1)
BaYaka	Early Childhood	0.01 (0, 0.01)	0.02 (0.02, 0.03)	0.04 (0.03, 0.05)	0.37 (0.35, 0.39)
BaYaka	Middle Childhood	0.02 (0.02, 0.02)	0.06 (0.06, 0.07)	0.11 (0.1, 0.11)	0.31 (0.3, 0.32)
BaYaka	Adolescence	0.03 (0.02, 0.03)	0.24 (0.23, 0.25)	0.12 (0.11, 0.12)	0.19 (0.18, 0.19)
Dukha	Early Childhood	0 (0, 0)	0.01 (0, 0.01)	0.02 (0.01, 0.02)	0.2 (0.19, 0.21)
Dukha	Middle Childhood	0.01 (0.01, 0.02)	0.01 (0.01, 0.02)	0.1 (0.08, 0.11)	0.1 (0.09, 0.12)
Dukha	Adolescence	0.01 (0.01, 0.01)	0.26 (0.25, 0.27)	0.14 (0.13, 0.15)	0.03 (0.02, 0.03)
Hadza	Early Childhood	0.05 (0.02, 0.09)	0.22 (0.16, 0.28)	0.04 (0.02, 0.08)	0.29 (0.22, 0.36)
Hadza	Middle Childhood	0.01 (0, 0.02)	0.25 (0.21, 0.29)	0.1 (0.07, 0.12)	0.12 (0.09, 0.15)
Hadza	Adolescence	0.03 (0.01, 0.07)	0.13 (0.08, 0.19)	0 (0, 0.03)	0.04 (0.01, 0.08)
Matsigenka	Early Childhood	0 (0, 0.01)	0.02 (0.02, 0.03)	0.02 (0.01, 0.02)	0.09 (0.08, 0.11)
Matsigenka	Middle Childhood	0.02 (0.02, 0.03)	0.11 (0.09, 0.12)	0.08 (0.07, 0.1)	0.14 (0.12, 0.16)
Matsigenka	Adolescence	0.04 (0.03, 0.05)	0.18 (0.15, 0.2)	0.2 (0.17, 0.22)	0.04 (0.03, 0.06)
Maya	Early Childhood	0.03 (0.03, 0.04)	0.03 (0.02, 0.03)	0.05 (0.04, 0.05)	0.58 (0.56, 0.59)
Maya	Middle Childhood	0.09 (0.08, 0.1)	0.08 (0.07, 0.09)	0.2 (0.19, 0.22)	0.22 (0.21, 0.24)
Maya	Adolescence	0.05 (0.04, 0.05)	0.12 (0.11, 0.13)	0.41 (0.39, 0.42)	0.01 (0, 0.01)
Mayangna	Early Childhood	0 (0, 0.01)	0 (0, 0.01)	0.03 (0.02, 0.03)	0.39 (0.37, 0.41)
Mayangna	Middle Childhood	0.04 (0.03, 0.04)	0.06 (0.05, 0.07)	0.19 (0.18, 0.2)	0.15 (0.14, 0.16)
Mayangna	Adolescence	0.02 (0.02, 0.03)	0.12 (0.11, 0.13)	0.33 (0.31, 0.35)	0.04 (0.03, 0.05)
Mikea	Early Childhood	0 (0, 0)	0.15 (0.13, 0.17)	0.02 (0.02, 0.03)	0.33 (0.31, 0.35)
Mikea	Middle Childhood	0.01 (0, 0.01)	0.26 (0.24, 0.27)	0.09 (0.08, 0.1)	0.21 (0.2, 0.22)
Mikea	Adolescence	0 (0, 0.01)	0.39 (0.36, 0.41)	0.11 (0.09, 0.13)	0.06 (0.05, 0.07)
Pumé	Early Childhood	0.01 (0, 0.01)	0.07 (0.06, 0.09)	0.05 (0.04, 0.06)	0.38 (0.36, 0.41)
Pumé	Middle Childhood	0.04 (0.03, 0.05)	0.18 (0.16, 0.19)	0.13 (0.12, 0.14)	0.16 (0.15, 0.18)
Pumé	Adolescence	0.05 (0.04, 0.05)	0.12 (0.1, 0.13)	0.29 (0.27, 0.3)	0.03 (0.02, 0.03)
Tsimane	Early Childhood	0.01 (0.01, 0.02)	0.02 (0.02, 0.02)	0.07 (0.07, 0.08)	0.26 (0.25, 0.27)
Tsimane	Middle Childhood	0.03 (0.03, 0.04)	0.06 (0.06, 0.07)	0.14 (0.13, 0.15)	0.19 (0.19, 0.2)
Tsimane	Adolescence	0.06 (0.05, 0.07)	0.09 (0.08, 0.1)	0.26 (0.24, 0.27)	0.05 (0.04, 0.06)
**Total**	**Early Childhood**	**0.01 (0.01, 0.01)**	**0.06 (0.06, 0.06)**	**0.04 (0.04, 0.04)**	**0.34 (0.33, 0.34)**
**Total**	**Middle Childhood**	**0.03 (0.03, 0.03)**	**0.11 (0.11, 0.11)**	**0.12 (0.12, 0.13)**	**0.22 (0.21, 0.22)**
**Total**	**Adolescence**	**0.03 (0.02, 0.03)**	**0.21 (0.2, 0.21)**	**0.19 (0.19, 0.2)**	**0.08 (0.08, 0.08)**

[1]Exact ages for Mikea children were not known. Children were instead categorized as early juveniles (5-8 years), late juveniles (9-15 years), and young adults (16-25 years), corresponding to early childhood, middle childhood, and adolescence, respectively, in the present table.

Table S4. Time allocation by society and gender. Binomial 90% confidence intervals are in parentheses. Values represent population proportions of observation time.

Society	Gender	Childcare	Food Production	Domestic Work	Play
Agta	Girls	0.06 (0.03, 0.1)	0.06 (0.03, 0.11)	0.02 (0, 0.05)	0.3 (0.23, 0.37)
Agta	Boys	0.03 (0.01, 0.05)	0.07 (0.04, 0.11)	0.01 (0, 0.03)	0.38 (0.32, 0.44)
Aka	Girls	0.02 (0.01, 0.02)	0.13 (0.12, 0.13)	0.1 (0.1, 0.11)	0.19 (0.18, 0.2)
Aka	Boys	0.01 (0.01, 0.01)	0.05 (0.05, 0.06)	0.05 (0.05, 0.06)	0.33 (0.32, 0.34)
Baka	Girls	0.02 (0.01, 0.02)	0.27 (0.26, 0.28)	0.1 (0.1, 0.11)	0.16 (0.15, 0.16)
Baka	Boys	0 (0, 0.01)	0.24 (0.23, 0.25)	0.08 (0.07, 0.09)	0.31 (0.3, 0.32)
BaYaka	Girls	0.04 (0.03, 0.04)	0.15 (0.15, 0.16)	0.15 (0.14, 0.16)	0.22 (0.21, 0.22)
BaYaka	Boys	0.01 (0.01, 0.01)	0.11 (0.11, 0.12)	0.07 (0.06, 0.07)	0.3 (0.29, 0.31)
Dukha	Girls	0.01 (0, 0.01)	0.04 (0.03, 0.05)	0.11 (0.1, 0.12)	0.06 (0.05, 0.07)
Dukha	Boys	0.01 (0, 0.01)	0.18 (0.17, 0.18)	0.08 (0.08, 0.09)	0.11 (0.1, 0.12)
Hadza	Girls	0.03 (0.02, 0.05)	0.24 (0.21, 0.28)	0.11 (0.08, 0.14)	0.23 (0.19, 0.26)
Hadza	Boys	0.01 (0, 0.03)	0.19 (0.15, 0.23)	0.01 (0, 0.03)	0.04 (0.02, 0.06)
Matsigenka	Girls	0.03 (0.02, 0.04)	0.06 (0.05, 0.07)	0.1 (0.09, 0.11)	0.07 (0.06, 0.08)
Matsigenka	Boys	0 (0, 0.01)	0.15 (0.13, 0.17)	0.07 (0.06, 0.08)	0.14 (0.13, 0.16)
Maya	Girls	0.07 (0.07, 0.08)	0.07 (0.06, 0.07)	0.3 (0.29, 0.31)	0.19 (0.18, 0.2)
Maya	Boys	0.02 (0.02, 0.03)	0.09 (0.08, 0.1)	0.07 (0.07, 0.08)	0.43 (0.41, 0.44)
Mayangna	Girls	0.04 (0.04, 0.05)	0.04 (0.03, 0.04)	0.25 (0.24, 0.26)	0.14 (0.13, 0.15)
Mayangna	Boys	0 (0, 0.01)	0.07 (0.06, 0.07)	0.09 (0.09, 0.1)	0.27 (0.26, 0.28)
Mikea	Girls	0 (0, 0.01)	0.17 (0.16, 0.19)	0.09 (0.08, 0.1)	0.24 (0.23, 0.26)
Mikea	Boys	0 (0, 0)	0.33 (0.32, 0.35)	0.05 (0.04, 0.06)	0.19 (0.18, 0.21)
Pumé	Girls	0.06 (0.05, 0.07)	0.06 (0.06, 0.07)	0.27 (0.26, 0.29)	0.06 (0.05, 0.06)
Pumé	Boys	0.01 (0.01, 0.02)	0.19 (0.17, 0.2)	0.09 (0.08, 0.1)	0.25 (0.23, 0.26)
Tsimane	Girls	0.05 (0.05, 0.06)	0.05 (0.04, 0.05)	0.17 (0.16, 0.17)	0.14 (0.13, 0.15)
Tsimane	Boys	0.01 (0.01, 0.01)	0.05 (0.05, 0.05)	0.09 (0.09, 0.1)	0.26 (0.25, 0.27)
**Total**	**Girls**	**0.04 (0.03, 0.04)**	**0.11 (0.11, 0.12)**	**0.16 (0.16, 0.17)**	**0.16 (0.16, 0.16)**
**Total**	**Boys**	**0.01 (0.01, 0.01)**	**0.13 (0.13, 0.13)**	**0.07 (0.07, 0.08)**	**0.26 (0.26, 0.27)**

Table S5. Dangerous Mammal Species present at each site, and their associated density (n/km2). Derived from the Pantheria database[51]. 1=Present, 0=Absent.

Species	n/km^2	Agta	Aka	Baka	BaYaka	Dukha	Hadza	Matsigenka	Maya	Mayangna	Mikea	Pume	Tsimane
Acinonyx jubatus	0.01	0	0	0	0	0	1	0	0	0	0	0	0
Canis lupus	0.01	0	0	0	0	1	0	0	0	0	0	0	0
Crocuta crocuta	0.13	0	0	0	0	0	1	0	0	0	0	0	0
Gorilla gorilla	0.59	0	1	1	1	0	0	0	0	0	0	0	0
Hippopotamus amphibius	4.87	0	1	1	1	0	1	0	0	0	0	0	0
Leopardus pardalis	0.43	0	0	0	0	0	0	1	0	0	0	0	0
Loxodonta africana	6.72	0	1	1	1	0	1	0	0	0	0	0	0
Panthera leo	0.11	0	0	0	0	0	1	0	0	0	0	0	0
Panthera onca	0.04	0	0	0	0	0	0	1	1	1	0	1	1
Panthera pardus	0.07	0	1	1	1	0	1	0	0	0	0	0	0
Puma concolor	0.02	0	0	0	0	0	0	1	1	1	0	1	0
Ursus arctos	0.02	0	0	0	0	1	0	0	0	0	0	0	0

Table S6. Medically Important Venomous Snake Species present at each site. Derived from Longbottom et al.[50]. 1=Present, 0=Absent.

Species	Agta	Aka	Baka	BaYaka	Dukha	Hadza	Matsigenka	Maya	Mayangna	Mikea	Pume	Tsimane
Agkistrodon bilineatus	0	0	0	0	0	0	0	1	0	0	0	0
Atheris broadleyi	0	0	1	0	0	0	0	0	0	0	0	0
Atheris squamigera	0	1	1	1	0	0	0	0	0	0	0	0
Atractaspis irregularis	0	1	1	1	0	0	0	0	0	0	0	0
Bitis arietans	0	1	1	0	0	1	0	0	0	0	0	0
Bitis gabonica	0	1	1	1	0	0	0	0	0	0	0	0
Bitis nasicornis	0	1	1	1	0	0	0	0	0	0	0	0
Bothriechis schlegelii	0	0	0	0	0	0	0	0	1	0	0	0
Bothrocophias hyoprora	0	0	0	0	0	0	0	0	0	0	0	1
Bothrocophias microphthalmus	0	0	0	0	0	0	1	0	0	0	0	0
Bothrops andianus	0	0	0	0	0	0	0	0	0	0	0	1
Bothrops asper	0	0	0	0	0	0	0	1	1	0	0	0
Bothrops atrox	0	0	0	0	0	0	0	0	0	0	0	1
Bothrops bilineatus	0	0	0	0	0	0	1	0	0	0	0	0
Bothrops mattogrossensis	0	0	0	0	0	0	0	0	0	0	0	1
Bothrops sanctaecrucis	0	0	0	0	0	0	0	0	0	0	0	1
Bothrops taeniatus	0	0	0	0	0	0	1	0	0	0	0	0
Crotalus durissus	0	0	0	0	0	0	1	0	0	0	1	1
Crotalus tzabcan	0	0	0	0	0	0	0	1	0	0	0	0
Dendroaspis angusticeps	0	0	0	0	0	1	0	0	0	0	0	0
Dendroaspis jamesoni	0	1	1	1	0	0	0	0	0	0	0	0
Dendroaspis polylepis	0	0	0	0	0	1	0	0	0	0	0	0
Dispholidus typus	0	1	1	0	0	1	0	0	0	0	0	0
Lachesis muta	0	0	0	0	0	0	1	0	0	0	0	1
Micrurus alleni	0	0	0	0	0	0	0	0	1	0	0	0
Micrurus lemniscatus	0	0	0	0	0	0	0	0	0	0	1	1
Micrurus multifasciatus	0	0	0	0	0	0	0	0	1	0	0	0
Micrurus nigrocinctus	0	0	0	0	0	0	0	0	1	0	0	0
Micrurus spixii	0	0	0	0	0	0	0	0	0	0	0	1
Micrurus spp	0	0	0	0	0	0	1	1	0	0	1	1
Micrurus surinamensis	0	0	0	0	0	0	0	0	0	0	0	1
Naja annulata	0	1	1	1	0	0	0	0	0	0	0	0
Naja haje	0	0	0	0	0	1	0	0	0	0	0	0
Naja melanoleuca	0	1	1	1	0	0	0	0	0	0	0	0
Naja nigricollis	0	0	0	0	0	1	0	0	0	0	0	0
Naja philippinensis	1	0	0	0	0	0	0	0	0	0	0	0
Ophiophagus hannah	1	0	0	0	0	0	0	0	0	0	0	0
Parias flavomaculatus	1	0	0	0	0	0	0	0	0	0	0	0
Porthidium nasutum	0	0	0	0	0	0	0	0	1	0	0	0
Porthidium yucatanicum	0	0	0	0	0	0	0	1	0	0	0	0
Pseudohaje goldii	0	1	1	1	0	0	0	0	0	0	0	0

Thelotornis kirtlandii	0	1	1	1	0	0	0	0	0	0	0	0
Tropidolaemus subannulatus	1	0	0	0	0	0	0	0	0	0	0	0
**Total**	**4**	**11**	**12**	**9**	**0**	**6**	**6**	**5**	**6**	**0**	**3**	**11**

Table S7. Correlations and Variance Inflation Factors (VIF) for variables in Model 3.

	Middle	Ado	Boys	NPP	Temp	Prec	Non-foraged	VIF
Middle Childhood		−0.51	−0.02	0.08	0.15	0.07	−0.13	1.39
Adolescence			−0.01	−0.16	−0.15	−0.09	0.01	1.39
Boys				−0.09	−0.13	−0.03	−0.02	1.03
Net Primary Productivity[1]					0.48	0.36	0.16	1.65
Annual Mean Temperature[1]						0.65	−0.37	2.67
Annual Precipitation[1]							−0.23	1.81
Prop Non-Foraged[1]								1.43

[1]*z*-score standardized

Table S8. Correlations and Variance Inflation Factors (VIF) for variables in Model 4.

	Middle	Ado	Boys	Mammal	Water	Non-foraged	VIF
Middle Childhood		−0.51	−0.02	0.07	0.07	−0.13	1.35
Adolescence			−0.01	0.02	−0.11	0.01	1.33
Boys				0.01	−0.05	−0.02	1.01
High Mammal Density					−0.39	−0.66	2.25
Water Quality/Quantity						0.11	1.27
Prop Non-Foraged[1]							1.95

[1]*z*-score standardized

Table S9. Correlations and Variance Inflation Factors (VIF) for variables in Model 5.

	Middle	Ado	Boys	Div	Non-foraged	VIF
Middle Childhood		−0.51	−0.02	−0.02	−0.13	1.39
Adolescence			−0.01	−0.04	0.01	1.37
Boys				−0.08	−0.02	1.01
Gendered Division of food production Labour					0.67	1.80
Prop Non-Foraged[1]						1.81

[1]*z*-score standardized

Table S10. Correlation of individual-level and society-level random effects for Model 1 (intercept only). The reported means are from the posterior samples (standard deviations in parentheses). Parameters in bold represent estimates whose 95% credible intervals do not cross zero. The top half of the matrix depicts correlations for individuals, the bottom half depicts correlations for societies.

	Childcare	Food production	Domestic work	Play
Childcare		**0.20 (0.05)**	**0.51 (0.04)**	**−0.17 (0.05)**
Food production	−0.14 (0.29)		**0.44 (0.04)**	**−0.22 (0.04)**
Domestic work	0.22 (0.29)	−0.08 (0.27)		**−0.36 (0.04)**
Play	0.14 (0.28)	0.25 (0.26)	0.12 (0.28)	

Table S11. Correlation of individual-level and society-level random effects for Model 2 (Individual-level variables). The reported means are from the posterior samples (standard deviations in parentheses). Parameters in bold represent estimates whose 95% credible intervals do not cross zero. The top half of the matrix depicts correlations for individuals, the bottom half depicts correlations for societies.

	Childcare	Food production	Domestic work	Play
Childcare		0.08 (0.06)	**0.24 (0.06)**	**0.22 (0.06)**
Food production	−0.09 (0.30)		**0.25 (0.05)**	0.08 (0.05)
Domestic work	0.25 (0.28)	0.03 (0.28)		**0.15 (0.05)**
Play	0.14 (0.28)	0.07 (0.27)	0.17 (0.27)	

Table S12. Mean standard deviations of the random effects from the posterior samples for Models 1-5. The standard deviations of these quantities from the posterior samples are in parentheses.

	Individual-level random effects					Societal-level random effects				
	Model 1	*Model 2*	*Model 3*	*Model 4*	*Model 5*	*Model 1*	*Model 2*	*Model 3*	*Model 4*	*Model 5*
Childcare	1.90 (0.10)	1.55 (0.08)	1.55 (0.08)	1.56 (0.08)	1.54 (0.08)	1.26 (0.46)	1.44 (0.57)	1.60 (0.72)	1.11 (0.39)	1.63 (0.65)
Food Production	1.64 (0.06)	1.36 (0.06)	1.36 (0.06)	1.36 (0.06)	1.36 (0.06)	0.91 (0.25)	0.79 (0.26)	0.61 (0.26)	0.81 (0.32)	0.81 (0.27)
Domestic work	1.23 (0.04)	0.86 (0.03)	0.86 (0.04)	0.86 (0.04)	0.86 (0.03)	0.66 (0.21)	0.66 (0.21)	0.57 (0.24)	0.72 (0.27)	0.72 (0.25)
Play	1.24 (0.05)	0.88 (0.03)	0.88 (0.03)	0.88 (0.03)	0.88 (0.03)	0.58 (0.17)	0.59 (0.17)	0.43 (0.16)	0.59 (0.18)	0.59 (0.18)

Table S13. Model comparisons for Models 1-5.

	WAIC	SE	dWAIC	dSE	pWAIC	Weight
Model 4	176042.9	448.17	0.0	NA	1750.1	0.81
Model 5	176045.9	447.97	3.0	4.38	1750.1	0.18
Model 2	176051.5	448.00	8.7	2.94	1751.1	0.01
Model 3	176052.3	448.09	9.5	2.88	1751.4	0.01
Model 1	176083.7	448.41	40.9	28.97	1842.4	0.00

Table S14. Posterior means of fixed effects for supplementary models investigating each environmental factor and ecological risk effect independently. Standard deviations are in parentheses. Parameters in bold represent estimates whose 95% credible intervals do not cross zero.

	Childcare	Food production	Domestic work	Play
Model 3.1—NPP				
Intercept	**−3.95 (0.69)**	**−3.01 (0.34)**	**−2.81 (0.28)**	**−1.06 (0.24)**
Boys	**−1.18 (0.27)**	0.06 (0.19)	−0.12 (0.14)	**0.66 (0.11)**
Middle	**1.33 (0.22)**	**0.93 (0.15)**	**1.50 (0.12)**	**−0.34 (0.10)**
Ado	**1.80 (0.23)**	**1.27 (0.17)**	**2.10 (0.13)**	**−2.19 (0.15)**
Boys*Middle	−0.41 (0.34)	0.27 (0.22)	**−0.66 (0.18)**	−0.22 (0.14)
Boys*Ado	**−1.19 (0.39)**	**0.54 (0.25)**	**−0.91 (0.19)**	**0.83 (0.20)**
Prop non-foraged[1]	0.10 (0.41)	−0.48 (0.24)	0.13 (0.20)	−0.13 (0.18)
NPP[1]	−0.10 (0.43)	0.11 (0.24)	−0.10 (0.20)	0.01 (0.18)
Model 3.2—Annual Mean Temperature				
Intercept	**−4.13 (0.67)**	**−3.05 (0.33)**	**−2.87 (0.27)**	**−1.16 (0.19)**
Boys	**−1.18 (0.28)**	0.04 (0.19)	−0.12 (0.14)	**0.67 (0.11)**
Middle	**1.33 (0.24)**	**0.93 (0.15)**	**1.50 (0.12)**	**−0.33 (0.11)**
Ado	**1.80 (0.25)**	**1.26 (0.17)**	**2.10 (0.13)**	**−2.17 (0.15)**
Boys*Middle	−0.41 (0.35)	0.27 (0.22)	**−0.67 (0.17)**	−0.23 (0.14)
Boys*Ado	**−1.19 (0.39)**	**0.56 (0.25)**	**−0.91 (0.19)**	**0.82 (0.20)**
Prop non-foraged[1]	0.18 (0.39)	−0.41 (0.23)	0.16 (0.19)	−0.04 (0.13)
Annual mean temp[1]	0.40 (0.46)	0.15 (0.28)	0.29 (0.22)	**0.39 (0.17)**
Model 3.3—Annual Precipitation				
Intercept	**−4.01 (0.71)**	**−3.07 (0.29)**	**−2.80 (0.29)**	**−1.07 (0.22)**
Boys	**−1.18 (0.27)**	0.05 (0.19)	−0.11 (0.13)	**0.65 (0.11)**
Middle	**1.32 (0.24)**	**0.93 (0.14)**	**1.50 (0.12)**	**−0.33 (0.11)**
Ado	**1.80 (0.24)**	**1.27 (0.17)**	**2.11 (0.13)**	**−2.18 (0.14)**
Boys*Middle	−0.40 (0.34)	0.28 (0.22)	**−0.67 (0.17)**	−0.22 (0.15)
Boys*Ado	**−1.19 (0.38)**	**0.55 (0.25)**	**−0.91 (0.18)**	**0.83 (0.20)**
Prop non-foraged[1]	0.11 (0.38)	**−0.47 (0.19)**	0.09 (0.18)	−0.12 (0.16)
Annual prec[1]	0.35 (0.35)	−0.23 (0.19)	0.09 (0.19)	0.13 (0.15)
Model 4.1— Water Quality/Quantity				
Intercept	**−2.59 (0.80)**	**−3.04 (0.64)**	**−2.26 (0.56)**	−0.80 (0.43)
Boys	**−1.14 (0.27)**	0.05 (0.19)	−0.11 (0.14)	**0.66 (0.11)**
Middle	**1.39 (0.23)**	**0.93 (0.15)**	**1.51 (0.12)**	**−0.32 (0.11)**
Ado	**1.87 (0.24)**	**1.27 (0.18)**	**2.11 (0.13)**	**−2.16 (0.14)**
Boys*Middle	−0.44 (0.35)	0.27 (0.22)	**−0.68 (0.17)**	−0.22 (0.14)
Boys*Ado	**−1.25 (0.38)**	**0.54 (0.25)**	**−0.92 (0.19)**	**0.82 (0.19)**
Prop non-foraged[1]	0.05 (0.36)	**−0.44 (0.22)**	0.08 (0.21)	−0.13 (0.17)
Water qual/quant	**−1.20 (0.42)**	0.04 (0.32)	−0.37 (0.29)	−0.19 (0.23)
Model 4.2— Dangerous Mammal Density				
Intercept	**−3.96 (0.57)**	**−2.97 (0.35)**	**−2.73 (0.28)**	**−1.13 (0.25)**
Boys	**−1.18 (0.27)**	0.04 (0.19)	−0.13 (0.14)	**0.67 (0.11)**
Middle	**1.33 (0.23)**	**0.93 (0.15)**	**1.49 (0.12)**	**−0.33 (0.11)**
Ado	**1.81 (0.24)**	**1.27 (0.18)**	**2.09 (0.13)**	**−2.18 (0.15)**
Boys*Middle	−0.41 (0.34)	0.26 (0.22)	**−0.66 (0.17)**	−0.22 (0.14)
Boys*Ado	**−1.19 (0.38)**	**0.55 (0.25)**	**−0.89 (0.19)**	**0.82 (0.19)**
Prop non-foraged[1]	−0.19 (0.36)	**−0.49 (0.24)**	−0.02 (0.20)	−0.06 (0.18)
Mammal density	−1.22 (0.67)	−0.26 (0.50)	−0.47 (0.42)	0.29 (0.39)

Boys: 1=boys, 0=girls. **Middle:** 1= Middle Childhood (between 7 and 12 years of age, late juveniles for the Mikea dataset), 0= Early Childhood (between 3 and 6 years of age, early juveniles for the Mikea dataset). **Ado:** 1=Adolescence (between 13 and 18 years of age, young adults for the Mikea dataset). **Prop non-foraged:** Proportion of domesticated foods and foods purchased and/or traded. **NPP:** Net Primary Productivity in gC/m^2/yr. **Annual mean temp:** Annual mean temperature, in °C. **Annual prec:** Annual Precipitation, in mm. **Mammal density:** Dangerous Mammal Density, Low/High. **Water qual/quant:** Water Quality/Quantity, four-point scale.

[1]These values were *z*-score standardized.

Table S15. Posterior means of fixed effects for supplementary models investigating environmental factors and Annual Mean Temperature excluding observations from Dukha. Standard deviations are in parentheses. Parameters in bold represent estimates whose 95% credible intervals do not cross zero.

	Childcare	Food production	Domestic work	Play
Model 3.5—Model 3 refit without Dukha Data				
Intercept	**−3.55 (0.86)**	**−2.94 (0.40)**	**−2.82 (0.25)**	**−1.04 (0.21)**
Boys	**−1.16 (0.27)**	0.01 (0.19)	−0.09 (0.14)	**0.73 (0.12)**
Middle	**1.32 (0.23)**	**0.93 (0.15)**	**1.51 (0.12)**	**−0.33 (0.11)**
Ado	**1.81 (0.24)**	**1.24 (0.18)**	**2.12 (0.13)**	**−2.18 (0.15)**
Boys*Middle	−0.44 (0.34)	0.30 (0.22)	**−0.71 (0.18)**	**−0.34 (0.16)**
Boys*Ado	**−1.43 (0.39)**	**0.56 (0.26)**	**−0.97 (0.20)**	**0.76 (0.21)**
Prop non-foraged[1]	0.28 (0.49)	−0.36 (0.27)	0.22 (0.16)	0.04 (0.17)
NPP[1]	−0.10 (0.59)	−0.02 (0.32)	−0.01 (0.20)	−0.11 (0.21)
Annual mean temp[1]	0.33 (0.50)	0.03 (0.27)	**0.34 (0.18)**	0.12 (0.17)
Annual prec[1]	0.11 (0.48)	−0.30 (0.25)	−0.18 (0.17)	−0.04 (0.16)
Model 3.7—Independent effect of Annual Mean Temperature without Dukha Data				
Intercept	**−3.78 (0.78)**	**−2.94 (0.33)**	**−2.81 (0.18)**	**−1.01 (0.19)**
Boys	**−1.19 (0.29)**	0.00 (0.20)	−0.10 (0.14)	**0.72 (0.12)**
Middle	**1.31 (0.23)**	**0.92 (0.14)**	**1.51 (0.13)**	**−0.33 (0.11)**
Ado	**1.79 (0.24)**	**1.22 (0.18)**	**2.11 (0.13)**	**−2.17 (0.15)**
Boys*Middle	−0.43 (0.34)	0.29 (0.23)	**−0.71 (0.18)**	**−0.34 (0.16)**
Boys*Ado	**−1.39 (0.40)**	**0.57 (0.26)**	**−0.96 (0.20)**	**0.76 (0.20)**
Prop non-foraged[1]	0.21 (0.40)	−0.43 (0.21)	0.20 (0.12)	−0.03 (0.13)
Annual mean temp[1]	0.40 (0.33)	−0.13 (0.17)	**0.26 (0.09)**	0.15 (0.11)

Boys: 1=boys, 0=girls. **Middle:** 1= Middle Childhood (between 7 and 12 years of age, late juveniles for the Mikea dataset), 0= Early Childhood (between 3 and 6 years of age, early juveniles for the Mikea dataset). **Ado:** 1=Adolescence (between 13 and 18 years of age, young adults for the Mikea dataset). **Prop non-foraged:** Proportion of domesticated foods and foods purchased and/or traded. **NPP:** Net Primary Productivity in gC/m^2/yr. **Annual mean temp:** Annual mean temperature, in °C. **Annual prec:** Annual Precipitation, in mm.

[1]These values were *z*-score standardized

Table S16. Posterior means of fixed effects for Models 3 and 4 refit excluding data from adolescents. Standard deviations are in parentheses. Parameters in bold represent estimates whose 95% credible intervals do not cross zero.

	Childcare	Food production	Domestic work	Play
		Model 3.6—Model 3 excluding adolescents		
Intercept	**−3.97 (0.74)**	**−3.14 (0.37)**	**−2.89 (0.28)**	**−1.09 (0.14)**
Boys	**−1.09 (0.30)**	0.14 (0.20)	−0.07 (0.14)	**0.63 (0.11)**
Middle	**1.53 (0.24)**	**0.76 (0.15)**	**1.44 (0.13)**	**−0.35 (0.10)**
Boys*Middle	−0.67 (0.36)	0.31 (0.24)	**−0.68 (0.17)**	−0.10 (0.14)
Prop non-foraged[1]	0.30 (0.43)	−0.47 (0.26)	0.21 (0.20)	0.15 (0.11)
NPP[1]	−0.27 (0.43)	0.12 (0.26)	−0.27 (0.20)	**−0.30 (0.10)**
Annual mean temp[1]	0.57 (0.51)	0.57 (0.31)	**0.49 (0.25)**	**0.56 (0.14)**
Annual prec[1]	0.28 (0.41)	−0.46 (0.24)	−0.11 (0.19)	−0.08 (0.10)
		Model 4.4—Model 4 excluding adolescents		
Intercept	**−2.43 (0.69)**	**−2.71 (0.76)**	**−2.20 (0.54)**	−0.56 (0.44)
Boys	**−1.05 (0.30)**	0.13 (0.20)	−0.07 (0.15)	**0.64 (0.11)**
Middle	**1.61 (0.25)**	**0.76 (0.15)**	**1.45 (0.13)**	**−0.34 (0.10)**
Boys*Middle	−0.71 (0.36)	0.32 (0.23)	**−0.67 (0.18)**	−0.10 (0.13)
Prop non-foraged[1]	−0.28 (0.33)	−0.60 (0.30)	−0.12 (0.24)	−0.08 (0.19)
Water qual/quant	**−1.18 (0.36)**	−0.11 (0.39)	−0.35 (0.27)	−0.30 (0.22)
Mammal density	**−1.39 (0.63)**	−0.35 (0.61)	−0.47 (0.47)	0.06 (0.41)

Boys: 1=boys, 0=girls. **Middle:** 1= Middle Childhood (between 7 and 12 years of age, late juveniles for the Mikea dataset), 0= Early Childhood (between 3 and 6 years of age, early juveniles for the Mikea dataset). **Ado:** 1=Adolescence (between 13 and 18 years of age, young adults for the Mikea dataset). **Prop non-foraged:** Proportion of domesticated foods and foods purchased and/or traded. **NPP:** Net Primary Productivity in $gC/m^2/yr$. **Annual mean temp:** Annual mean temperature, in °C. **Annual prec:** Annual Precipitation, in mm. **Mammal density:** Dangerous Mammal Density, Low/High. **Water qual/quant:** Water Quality/Quantity, four-point scale.

[1]These values were *z*-score standardized.

Table S17. Posterior means of fixed effects for supplementary models investigating the effects of additional correlates for cross-cultural variation in time allocation. These are Coefficient of Variation of Monthly Precipitation (CV %) and the prevalence of medically important venomous snakes. Standard deviations are in parentheses. Parameters in bold represent estimates whose 95% credible intervals do not cross zero.

	Childcare	Food production	Domestic work	Play
Model 3.4—Coefficient of Variation of Monthly Precipitation				
Intercept	**−3.93 (0.68)**	**−3.17 (0.28)**	**−2.81 (0.31)**	**−1.03 (0.21)**
Boys	**−1.18 (0.27)**	0.02 (0.19)	−0.13 (0.14)	**0.66 (0.11)**
Middle	**1.33 (0.23)**	**0.92 (0.15)**	**1.50 (0.12)**	**−0.32 (0.10)**
Ado	**1.80 (0.24)**	**1.25 (0.18)**	**2.10 (0.13)**	**−2.17 (0.15)**
Boys*Middle	−0.40 (0.34)	0.30 (0.22)	**−0.66 (0.17)**	−0.22 (0.14)
Boys*Ado	**−1.19 (0.39)**	**0.57 (0.26)**	**−0.90 (0.19)**	**0.82 (0.20)**
Prop non-foraged[1]	0.08 (0.40)	**−0.41 (0.17)**	0.08 (0.19)	−0.14 (0.14)
CV %[1]	−0.38 (0.39)	0.29 (0.18)	−0.03 (0.20)	−0.22 (0.15)
Model 4.3—Medically Important Venomous Snakes				
Intercept	**−3.96 (0.73)**	**−3.02 (0.36)**	**−2.74 (0.33)**	**−0.98 (0.23)**
Boys	**−1.18 (0.27)**	0.02 (0.19)	−0.13 (0.13)	**0.67 (0.11)**
Middle	**1.34 (0.23)**	**0.92 (0.14)**	**1.49 (0.12)**	**−0.32 (0.10)**
Ado	**1.82 (0.24)**	**1.26 (0.18)**	**2.10 (0.13)**	**−2.16 (0.15)**
Boys*Middle	−0.40 (0.34)	0.29 (0.21)	**−0.66 (0.17)**	−0.23 (0.14)
Boys*Ado	**−1.20 (0.39)**	**0.56 (0.25)**	**−0.90 (0.19)**	**0.81 (0.20)**
Prop non-foraged[1]	0.09 (0.38)	−0.44 (0.23)	0.10 (0.20)	−0.11 (0.15)
Snakes[1]	0.17 (0.45)	−0.04 (0.28)	0.15 (0.23)	0.27 (0.18)

Boys: 1=boys, 0=girls. **Middle:** 1= Middle Childhood (between 7 and 12 years of age, late juveniles for the Mikea dataset), 0= Early Childhood (between 3 and 6 years of age, early juveniles for the Mikea dataset). **Ado:** 1=Adolescence (between 13 and 18 years of age, young adults for the Mikea dataset). **Prop non-foraged:** Proportion of domesticated foods and foods purchased and/or traded. **NPP:** Net Primary Productivity in $gC/m^2/yr$. **CV %:** Coefficient of Variation of Monthly Precipitation (%). **Snakes:** Number of Medically Important Venomous Snake Species at each site.

[1]These values were *z*-score standardized

4. Supplementary Figures

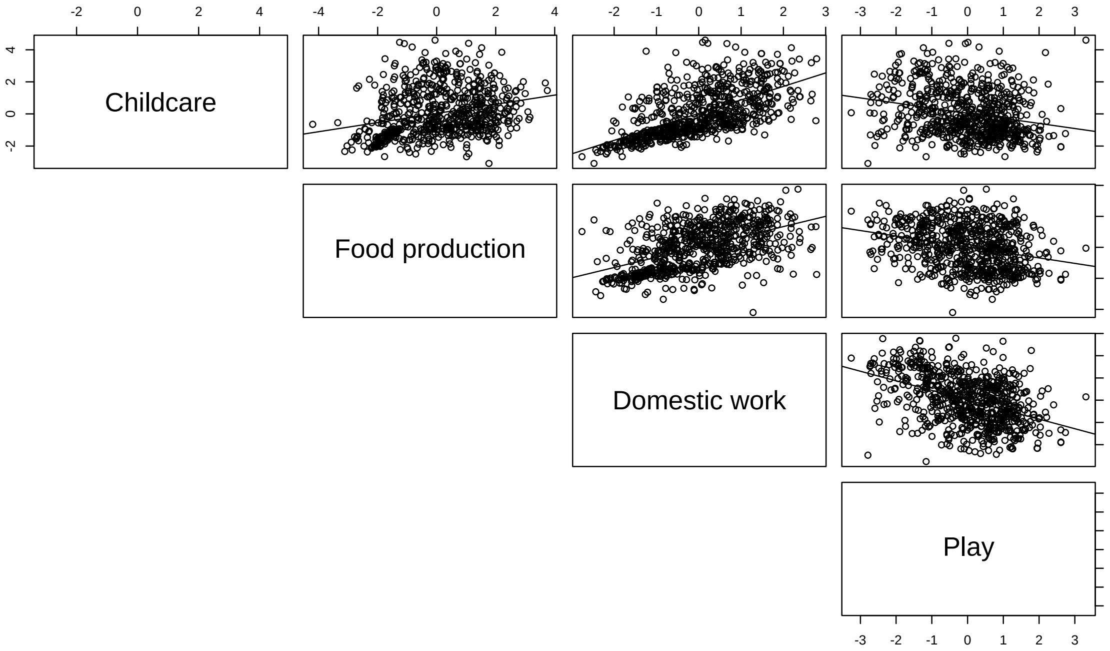

Figure S1. Correlation of individual random effects from Model 1. Values are medians of the posterior samples for each activity.

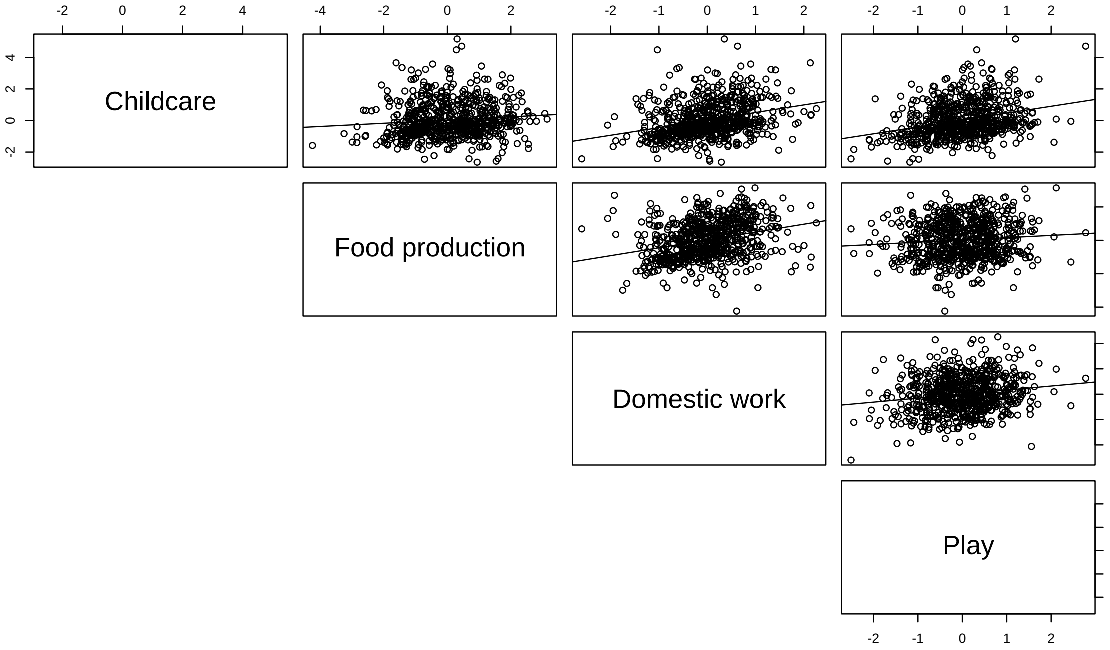

Figure S2. Correlation of individual random effects from Model 2. Values are medians from the posterior samples for each activity.

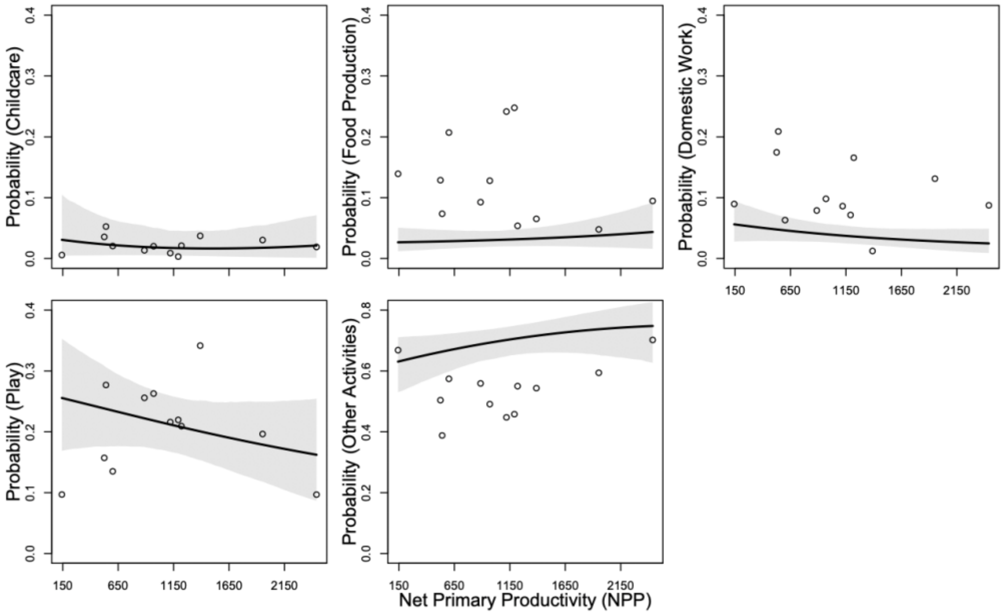

Figure S3. Model 3 predictions for Childcare, Food Production, Domestic Work, Play, and Other Activities (reference) as a function of Net Primary Productivity (NPP). Proportion Non-Foraged Foods, Annual Precipitation, and Annual Mean Temperature are held at the sample mean. Age and Gender are held at ‘early childhood’ and ‘girls’ respectively. Shaded area represents 89th percentile credible intervals, as calculated from the posterior samples. Scatterplots of children’s proportional time allocation to each activity by society are overlaid.

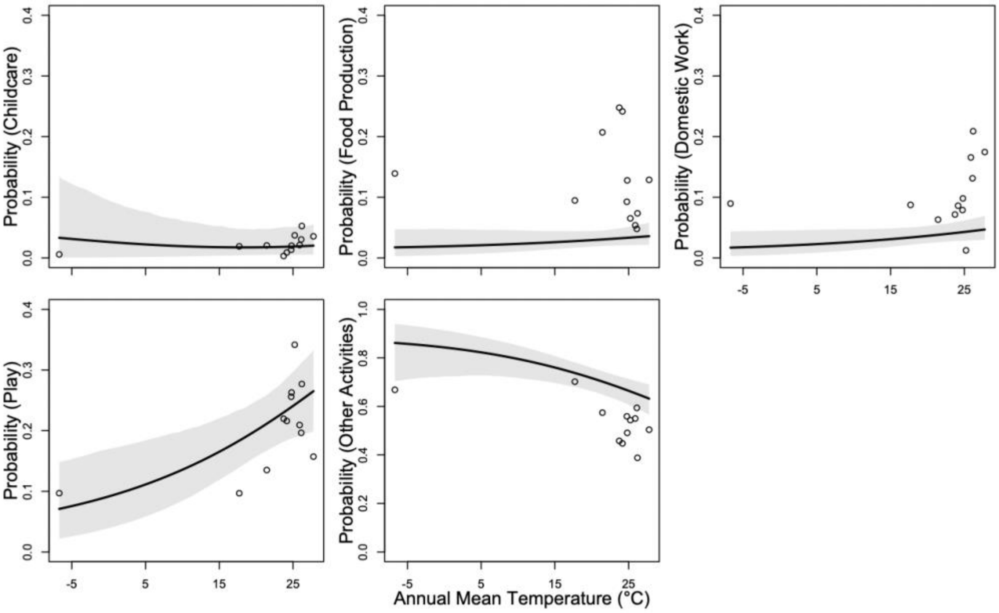

Figure S4. Model 3 predictions for Childcare, Food Production, Domestic Work, Play, and Other Activities (reference) as a function of Annual Mean Temperature. Proportion Non-Foraged Foods, Annual Precipitation, and Net Primary Productivity are held at the sample mean. Age and Gender are held at 'early childhood' and 'girls' respectively. Shaded area represents 89th percentile credible intervals, as calculated from the posterior samples. Scatterplots of children's proportional time allocation to each activity by society are overlaid.

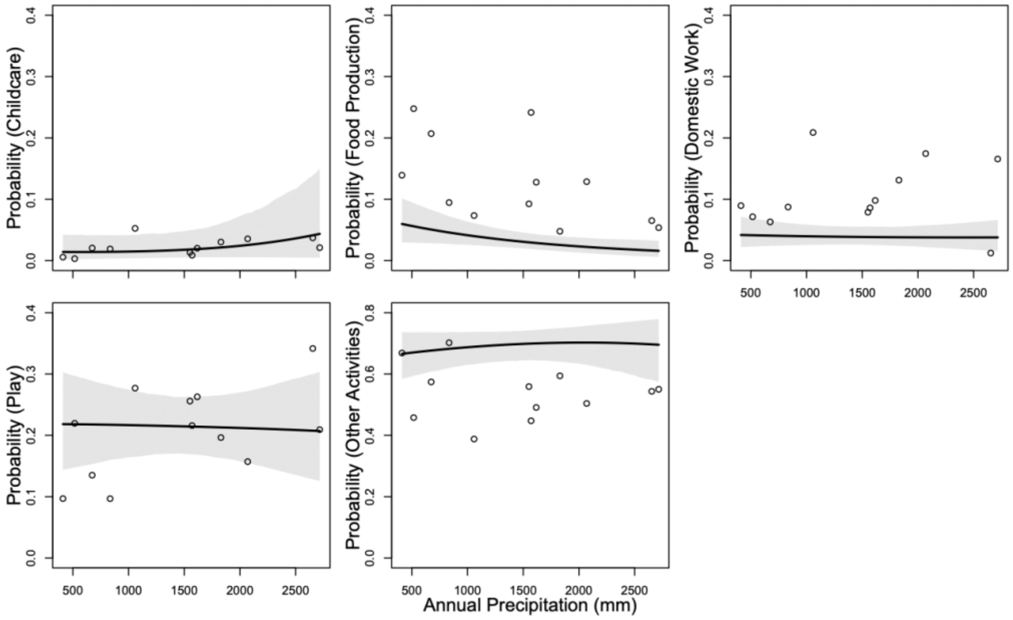

Figure S5. Model 3 predictions for Childcare, Food Production, Domestic Work, Play, and Other Activities (reference) as a function of Annual Precipitation. Proportion Non-Foraged Foods, Net Primary Productivity, and Annual Mean Temperature are held at the sample mean. Age and Gender are held at 'early childhood' and 'girls' respectively. Shaded area represents 89th percentile credible intervals, as calculated from the posterior samples. Scatterplots of children's proportional time allocation to each activity by society are overlaid.

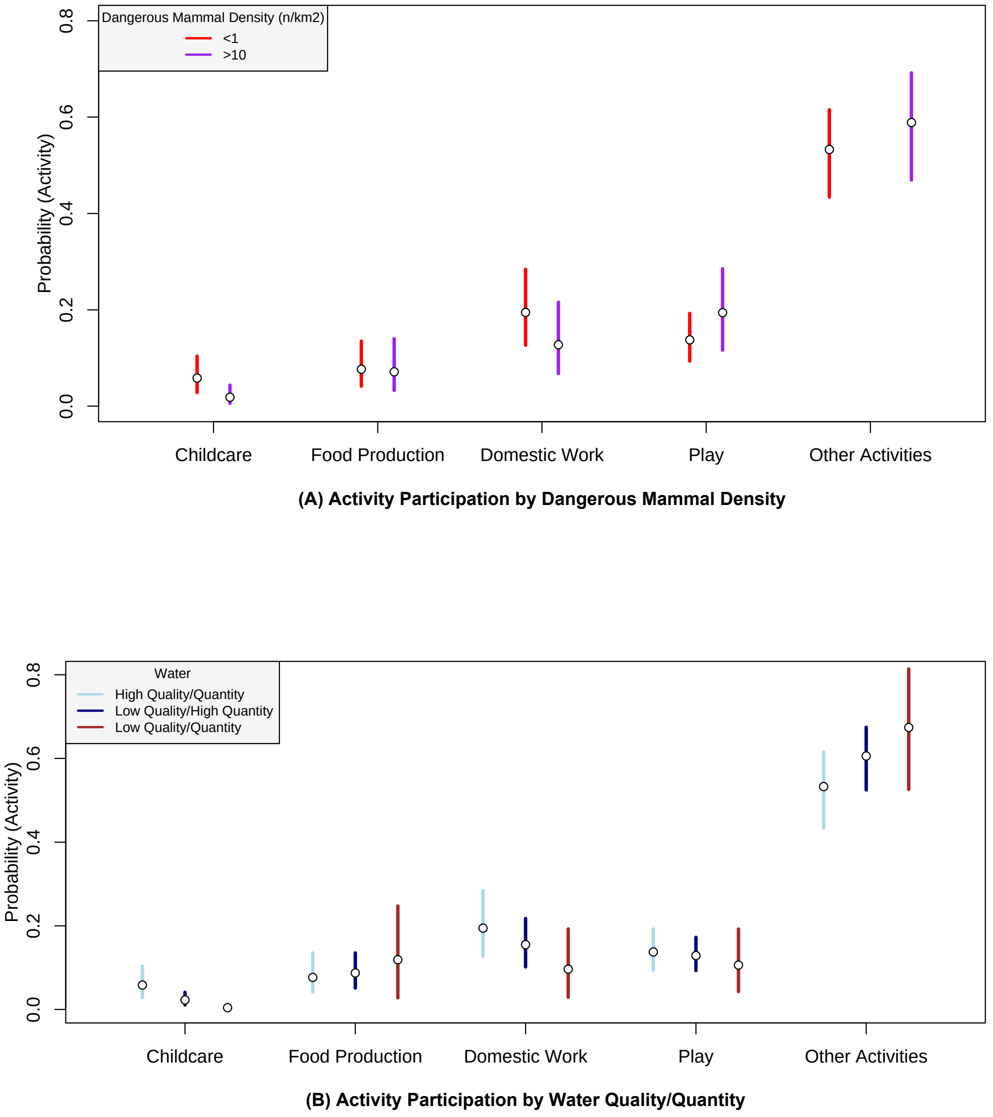

Figure S6. Model 4 predictions for the probability that a child engages in Childcare, Food Production, Domestic Work, Play, and Other Activities (reference) a function of (A) Dangerous Mammal Density and (B) Water Quality/Quantity for children in middle childhood. Proportion Non-Foraged Food is held at the sample mean. Gender is held at its reference value. Water Quality/Quantity and Dangerous Mammal Density are held constant at the reference value (low risk) in A and B respectively. Intervals represent 89th percentile credible intervals, as calculated from the posterior samples.

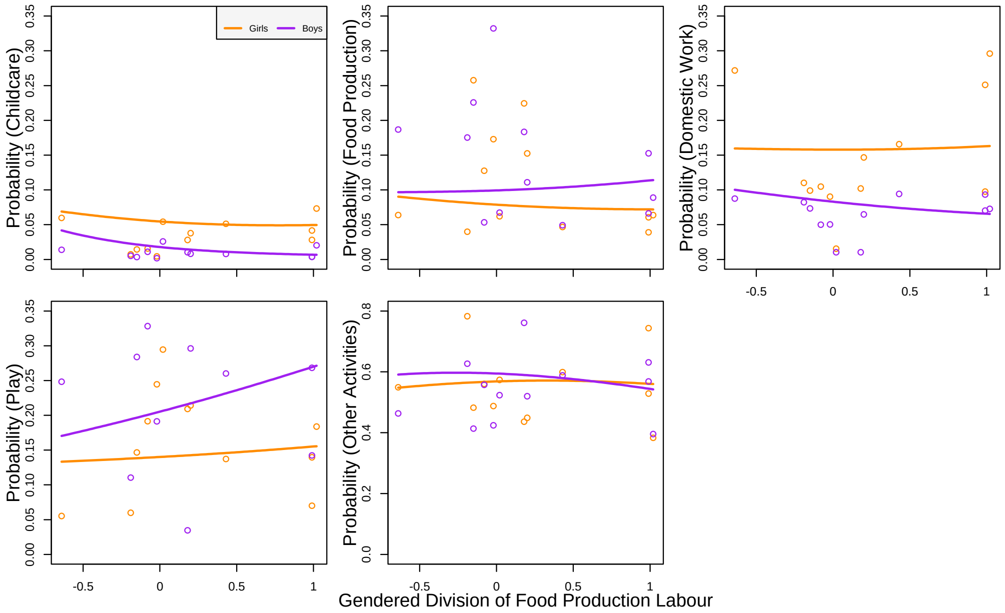

Figure S7. Model 5 predictions for the probability children engage in Childcare, Food Production, Domestic Work, Play and Other Activities (reference) as a function of Gendered Division of Food Production Labour and Gender. Age is held at middle childhood. Proportion Non-Foraged Food is held at the sample mean. Shaded areas represent 89th percentile credible intervals, as calculated from the posterior samples. Scatterplots of children's proportional time allocation to each activity by society are overlaid.

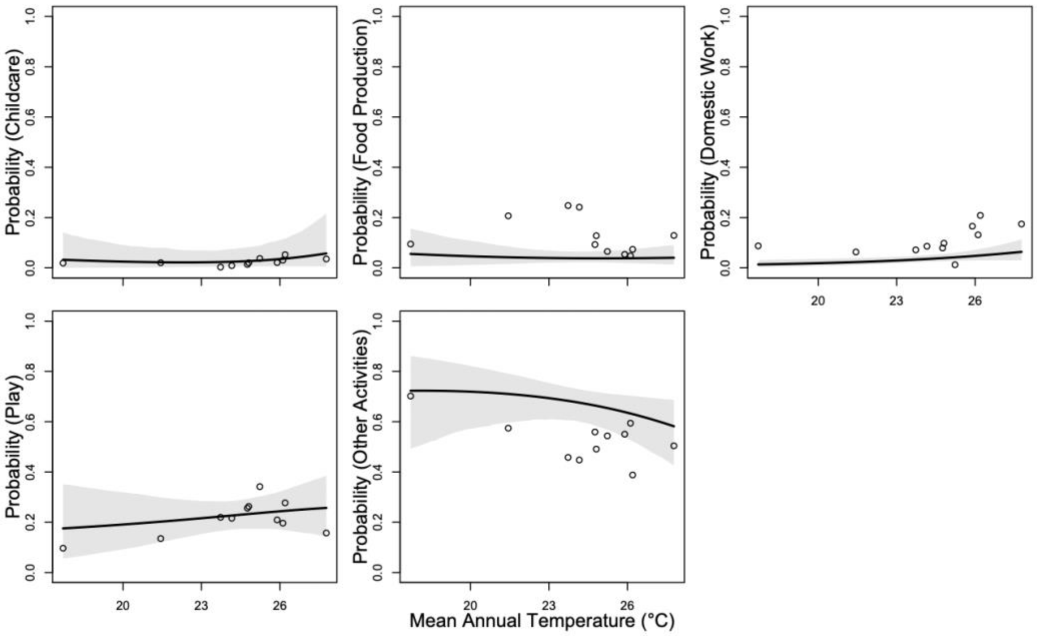

Figure S8. Model 3.5 predictions refitting Model 3 excluding the Dukha data for Childcare, Food Production, Domestic Work, Play, and Other Activities (reference) as a function of Annual Mean Temperature. Proportion Non-Foraged Foods, Annual Precipitation, and Net Primary Productivity are held at the sample mean. Age and Gender are held at 'early childhood' and 'girls' respectively. Shaded area represents 89th percentile credible intervals, as calculated from the posterior samples. Scatterplots of children's proportional time allocation to each activity by society are overlaid.

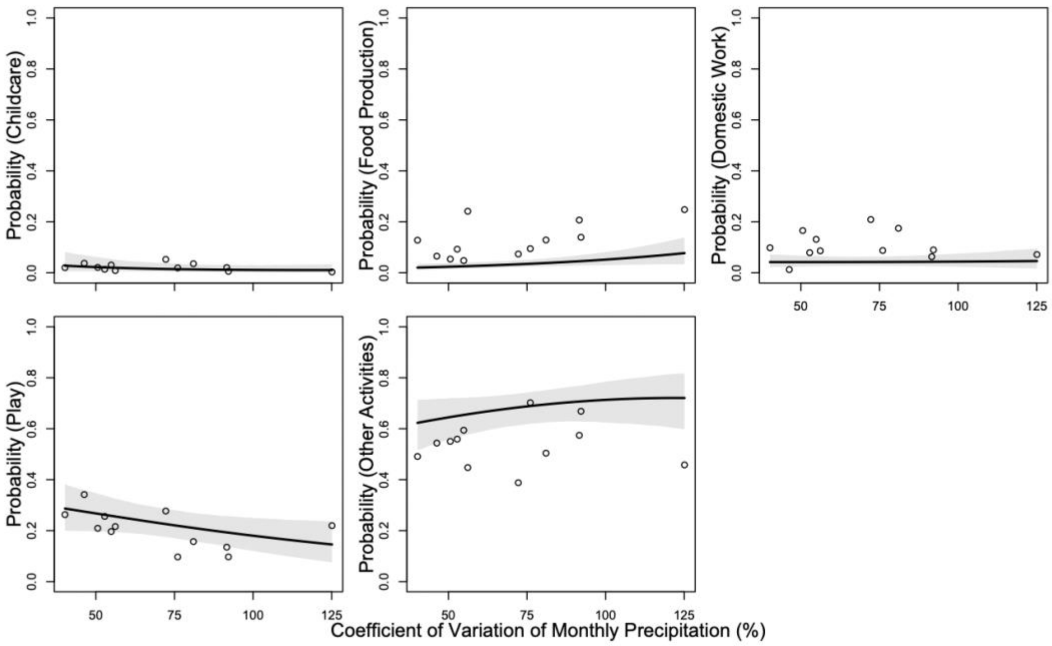

Figure S9. Model 3.4 predictions for Childcare, Food Production, Domestic Work, Play, and Other Activities (reference) as a function of Coefficient of Variation of Monthly Precipitation (CV %). Proportion Non-Foraged Foods is held at the sample mean. Age and Gender are held at 'early childhood' and 'girls' respectively. Shaded area represents 89th percentile credible intervals, as calculated from the posterior samples. Scatterplots of children's proportional time allocation to each activity by society are overlaid.

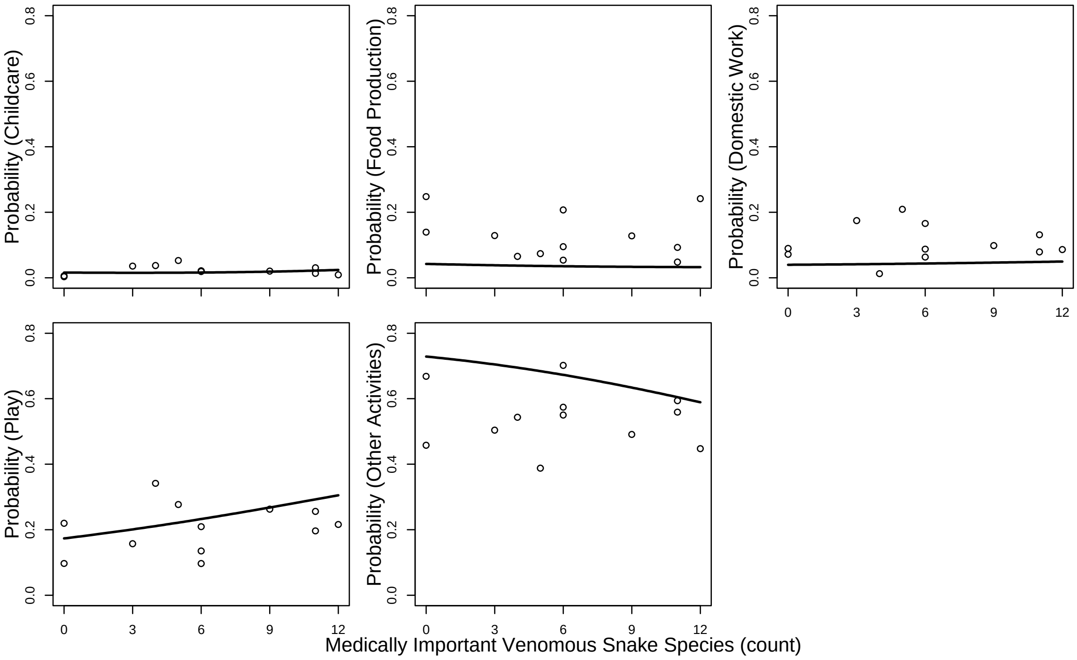

Figure S10. Model 4.3 predictions for Childcare, Food Production, Domestic Work, Play, and Other Activities (reference) as a function of total number of Medically Important Venomous Snake species. Proportion Non-Foraged Foods is held at the sample mean. Age and Gender are held at 'early childhood' and 'girls' respectively. Shaded area represents 89th percentile credible intervals, as calculated from the posterior samples. Scatterplots of children's proportional time allocation to each activity by society are overlaid.

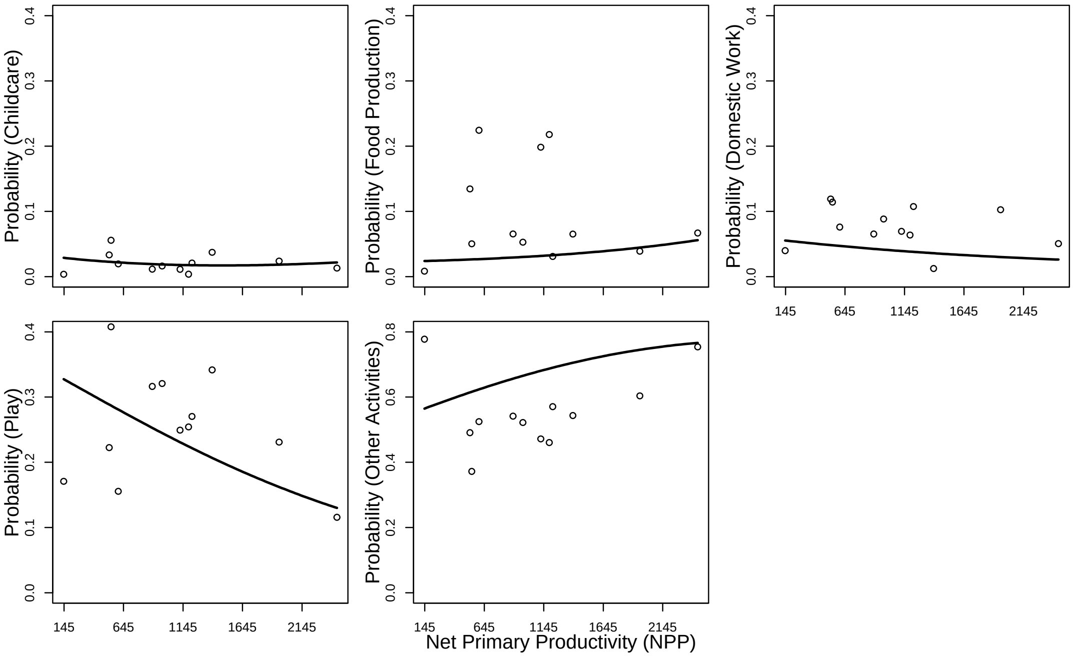

Figure S11. Model 3.6 predictions refitting Model 3 excluding adolescents for Childcare, Food Production, Domestic Work, Play, and Other Activities (reference) as a function of Net Primary Productivity (NPP). Proportion Non-Foraged Foods, Annual Precipitation, and Annual Mean Temperature are held at the sample mean. Age and Gender are held at ‘early childhood’ and ‘girls’ respectively. Shaded area represents 89th percentile credible intervals, as calculated from the posterior samples. Scatterplots of children’s proportional time allocation to each activity by society are overlaid.

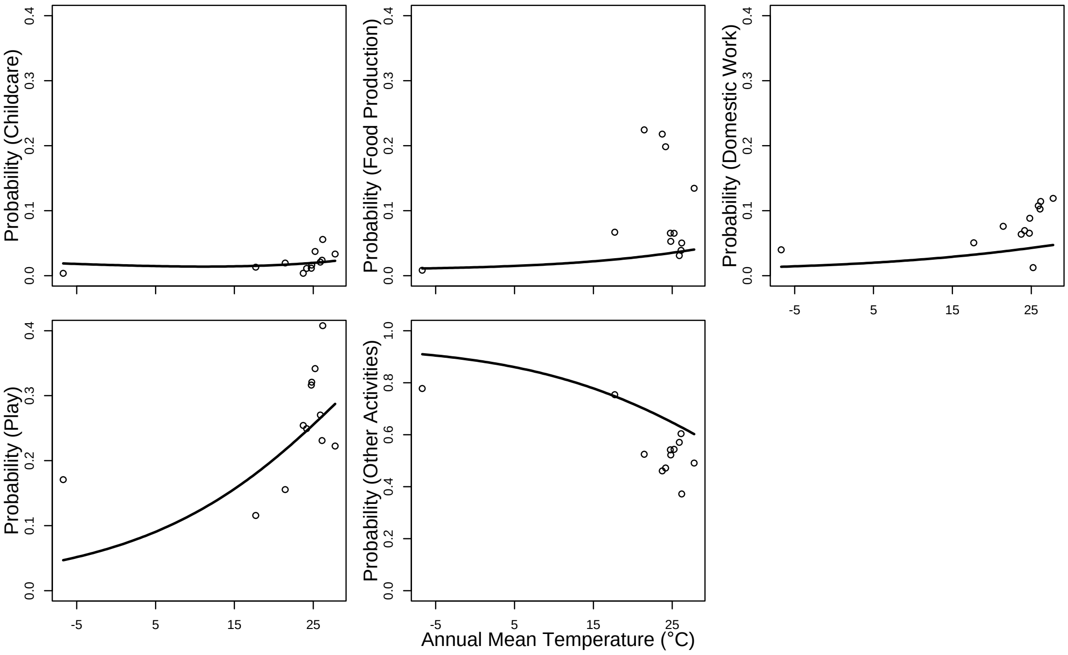

Figure S12. Model 3.6 predictions refitting Model 3 excluding adolescents for Childcare, Food Production, Domestic Work, Play, and Other Activities (reference) as a function of Annual Mean Temperature. Proportion Non-Foraged Foods, Annual Precipitation, and Net Primary Productivity are held at the sample mean. Age and Gender are held at ‘early childhood’ and ‘girls’ respectively. Shaded area represents 89th percentile credible intervals, as calculated from the posterior samples. Scatterplots of children’s proportional time allocation to each activity by society are overlaid.

5. Site-specific acknowledgements

BaYaka. Funding for fieldwork in the Republic of Congo was provided to SLL by the Cambridge International Trust, the Social Sciences and Humanities Research Council of Canada Doctoral Scholarship (752-2016-0555), the Ruggles-Gates Fund for Biological Anthropology from the Royal Anthropological Institute of Great Britain and Ireland, the Smuts Memorial Fund, the Worts Travelling Grant, and the Cambridge School of Biological Sciences Fieldwork Fund. SLL would like to thank the BaYaka families who participated in the research, Prof. Clobite Bouka-Biona from the Institut de Recherche en Sciences Exactes et Naturelles, who facilitated the acquisition of research permits and infrastructure, DZABATOU Moise, who served as a community liaison, and MEKOUNO Paul, who assisted in data collection.

Aka. Funding for fieldwork in the Central African Republic was provided to AHB by the National Science Foundation (DGE-0549425), and the Wenner-Gren Foundation Dissertation Fieldwork Grant (GR 8021). AHB would like to thank Mboulou Aubin and Mboula Edward for assistance during data collection, and the Aka families who made this research possible.

Hadza. ANC acknowledges the Hadza for their long-standing participation in, and patience with, research inquiries, and Frank Marlowe for his contributions to the data collection in 2005, whose grant (NSF #0242455) funded part of the data collection in Tanzania.

Agta. Funding for fieldwork in the Philippines was provided to RVH by Tropenbos International and Mabuwaya Foundation Inc. RVH would also like to thank her research assistant, Melody Capal, and her collaborators, Tessa Minter, Merlijn van Weerd, and Jan van der Ploeg, in addition to the communities who participated in the research.

Dukha. Funding for fieldwork in Mongolia was provided to TAS, MJO, and RH by the National Science Foundation's Arctic Social Sciences Program (PLR 1442166), a Fulbright Scholar Flex Research Grant (#48140555), the University of Wyoming's Department of Anthropology, George C. Frison Institute of Archaeology and Anthropology, and Research Office. TAS, MJO, and RH would like to thank the Dukha people; without their consent, support, help, and kindness, this work would not have been possible.

Baka. Funding for fieldwork in Cameroon was provided to KS by a JSPS Grant-in-Aid for Scientific Research for the project 'A Study of Human Learned Behavior Based on Fieldwork among Hunter-Gatherers' (Grant No. 22101003; primary investigator Prof. Dr. Hideaki Terashima) and the K. Matsushita Foundation (Grant No. 12-187; chief researcher Koji Sonoda). KS would like to acknowledge and sincerely thank the Baka children for allowing him into their daily lives.

Savannah Pumé and Maya. Funding for fieldwork in Mexico and Venezuela was provided to KLK by the National Science Foundation (0349963 and DBS-9123875), the National Institutes of Health (AG19044-01), the L.S.B. Leakey Foundation (awarded to Russell Greaves), the Milton Fund, and Harvard University. KLK is grateful to the Savanna Pumé and Maya who have graciously allowed her to observe them at work and play for many years.

Mayangna. Funding for fieldwork in Nicaragua was provided to JK by a Fulbright student grant, the National Science Foundation (Dissertation Improvement Award #0413037), the Hill

Foundation, and a William Sanders dissertation grant. Josiah Townsend provided information on the snakes that are found in this setting.

Tsimane. Funding to support research in Bolivia was provided through the NIH and NSF. JS and HED thank the Tsimane for participating, and THLHP personnel for providing logistical support. JS and HED also thank Stacey Rucas, Jeff Winking, Amanda Veile, Lisa Levenson, Sara Mulville, and Chris von Rueden for collecting behavioural data.

Mikea. Funding for fieldwork in Madagascar was provided to BT by a Fulbright IIE, and by an NSF DDRIG (BCS-9808984) with faculty co-PI Bruce Winterhalder. BT would like to thank Tsiazonera, Jaovola Tombo, and Tsimitamby for assistance in data collection.

6. References

1. Headland, T. N. Thirty endangered languages in the Philippines. *Work Pap. Summer Inst. Linguist. Univ. North Dakota Sess.* **47**, (2003).
2. Minter, T. The Agta of the Northern Sierra Madre. Livelihood Strategies and Resilience among Philippine Hunter-Gatherers. (University of Leiden, 2010).
3. Goodman, M. J., Bion Griffin, P. & Estioko-Griffin, A. A. The Compatibility of Hunting and Mothering among the Agta Hunter-Gatherers of the Philippines. *Sex Roles* **1212**, 1199–1209 (1985).
4. Estioko-Griffin, A. A. & Bion Griffin, P. Woman the Hunter: The Agta. in *Woman the Gatherer* (ed. Dalhberg, F.) 121–152 (Yale University Press, 1981).
5. Estioko-Griffin, A. A. Women as Hunters: The Case of an Eastern Cagayan Agta Group. in *The Agta of Northeastern Luzon* (eds. Bion Griffin, P. & Estioko-Griffin, A. A.) 18–32 (San Carlos Publications, 1985).
6. Page, A. E. *et al.* Children are important too: juvenile playgroups and maternal childcare in a foraging population, the Agta. *OSF Prepr.* doi:10.31219/osf.io/8xuj2.
7. Hagen, R., Ploeg, J. Van Der & Minter, T. How do hunter-gatherers learn?: The transmission of indigenous knowledge among the Agta of the Philippines. *Hunt. Gatherer Res.* **2**, 389–413 (2016).
8. Bahuchet, S. Food supply uncertainty among the Aka pygmies (Lobaye, Central African Republic). in *Coping With Uncertainty in Food Supply* (eds. De Garine, I. & Harrison, G.) 118–149 (Oxford University Press, 1988).
9. Kitanishi, K. Seasonal Changes in the Subsistence Activities and Food Intake of the Aka Hunter-Gatherers in Northeastern Congo. *Afr. Study Monogr.* **16**, 73–118 (1995).
10. Kitanishi, K. Food sharing among the Aka hunter-gatherers in northeastern Congo. *Afr. Study Monogr.* **Suppl. 25**, 3–32 (1998).
11. Altmann, J. Observational study of behaviour: sampling methods. *Behaviour* **49**, 227–267 (1974).
12. Boyette, A. H. Children's play and culture learning in an egalitarian foraging society. *Child Dev.* **87**, 759–769 (2016).
13. Boyette, A. H. Children's play and the integration of social and individual learning: A cultural niche construction perspective. in *Social learning and innovation in contemporary hunter-gatherers: Evolutionary and ethnographic perspectives* (eds. Terashima, H. & Hewlett, B. S.) 159–169 (Springer Japan, 2016).
14. Boyette, A. H. & Hewlett, B. S. Autonomy, Equality and Teaching among Aka Foragers and Ngandu Farmers of the Congo Basin. *Hum. Nat.* **28**, 289–322 (2017).
15. Lew-Levy, S. & Boyette, A. H. Evidence for the adaptive learning function of work and work-themed play among Aka Forager and Ngandu farmer children from the Congo Basin. *Hum. Nat.* **29**, 157–185 (2018).
16. Hagino, I. & Yamauchi, T. High Motivation and Low Gain: Food Procurement from Rainforest Foraging by Baka Hunter-Gatherer Children. in *Social Learning and Innovation in Contemporary Hunter-Gatherers: Evolutionary and Ethnographic Perspectives* (eds. Terashima, H. & Hewlett, B. S.) 135–146 (Springer Japan, 2016).
17. Gallois, S., Duda, R., Hewlett, B. S. & Reyes-garcía, V. Children's Daily Activities and Knowledge Acquisition: A case Study among the Baka from Southeastern Cameroon. *J. Ethnobiol. Ethnomed.* **11**, 86–99 (2015).
18. Gallois, S., Duda, R. & Reyes-Garcia, V. Local ecological knowledge among Baka children: A case of 'children's culture'? *J. Ethnobiol.* **37**, 60–80 (2017).
19. Kamei, N. An educational project in the forest: Schooling for the Baka children in Cameroon. *Afr. Study Monogr.* **26**, 185–195 (2001).
20. Lewis, J. Forest Hunter-Gatherers and Their World: A Study of Mbendjele Yaka

Pygmies of Congo-Brazzaville and Their Secular and Religious Activities and Representations. (London School of Economics and Political Science, 2002).
21. Joiris, D. V. The framework of Central African hunter-gatherers and neighbouring societies. *African Study Monogr. Suppl. Issue* **Suppl.28**, 57–79 (2003).
22. Hewlett, B. S. *Intimate fathers: The nature and context of Aka Pygmy paternal infant care*. (University of Michigan Press, 1991).
23. Thomas, J. M. & Bahuchet, S. *Encyclopédie des Pygmée Aka: Techniques, Langage et Société de Chasseurs-Cueilleurs de la Forêt Centrafricaine (Sud-Centrafrique et Nord-Congo)*. (Peeters Press, 1991).
24. Boyette, A. H. & Lew-Levy, S. Socialization, autonomy and cooperation: Insights from task assignments among Congolese BaYaka children. *Ethos*.
25. Lew-Levy, S., Boyette, A. H., Crittenden, A. N., Hewlett, B. S. & Lamb, M. E. Gender-typed and gender-segregated play among Tanzanian Hadza and Congolese BaYaka hunter-gatherer children and adolescents. *Child Dev.* **91**, 1284–1301 (2020).
26. Lew-levy, S. *et al.* Who teaches children to forage? Exploring the primacy of child-to-child teaching among the Hadza and BaYaka hunter-gatherers of Tanzania and Congo. *Evol. Hum. Behav.* **40**, 12–22 (2020).
27. Lew-Levy, S. *et al.* Inter- and intra-cultural variation in learning-through-participation among Hadza and BaYaka forager children and adolescents from Tanzania and Congo. *J. Psychol. Africa* **29**, 309–318 (2019).
28. Crittenden, A. N., Conklin-Brittain, N. L., Zes, D. A., Schoeninger, M. J. & Marlowe, F. W. Juvenile Foraging among the Hadza: Implications for Human Life History. *Evol. Hum. Behav.* **34**, 299–304 (2013).
29. Pollom, T. R., Herlosky, K. N., Mabulla, I. A. & Crittenden, A. N. Changes in juvenile foraging behavior: Preliminary evidence of early nutrition transition among Hadza foragers. *Hum. Nat.* (2020).
30. Johnson, O. R. & Johnson, A. male/female relations and the organization of work in a Machiguenga community. *Am. Ethnol.* **2**, 634–648 (1975).
31. Johnson, A. & Johnson, O. R. *Time allocation among the Machiguenga of Shimaa*. (Human Relations Area Files, 1987).
32. Baksh, M. *Time allocation among the Machiguenga of Camana*. *Cross-Cultural Studies in Time Allocation* (Human Relations Area Files, 1990).
33. Johnson, A. *Families of the Forest: The Matsigenka Indians of the Peruvian Amazon*. (University of California Press, 2003).
34. Johnson, A. Time allocation in a Machiguenga community. *Ethnology* **14**, 301–310 (1975).
35. Kramer, K. L. *Maya Children: Helpers at the Farm.* (Harvard University Press, 2005).
36. Koster, J., Grote, M. N. & Winterhalder, B. Effects on Household Labor of Temporary Out-migration by Male Household Heads in Nicaragua and Peru: An Analysis of Spot-check Time Allocation Data Using Mixed-effects Models. *Hum. Ecol.* **41**, 221–237 (2013).
37. Tucker, B. *et al.* Ethnic Markers without Ethnic Conflict: Why do Interdependent Masikoro, Mikea, and Vezo of Madagascar Signal their Ethnic Differences? *Hum. Nat.* **32**, 529–556 (2021).
38. Kramer, K. L. & Greaves, R. D. Diversity or Replace. What happens to wild foods when cultigens are introduced into hunter-gatherer diets. in *Why Forage? Hunters and Gatherers Living in the 21st Century* (eds. Codding, B. & Kramer, K. L.) 15–42 (School of Advanced Research, 2016).
39. Stieglitz, J., Trumble, B. C., Kaplan, H. & Gurven, M. Marital violence and fertility in a relatively egalitarian high-fertility population. *Nature Human Behaviour* vol. 2 565–

572 (2018).
40. Gurven, M. *et al.* The Tsimane Health and Life History Project: Integrating anthropology and biomedicine. *Evol. Anthropol.* **26**, 54–73 (2017).
41. Bock, J. & Johnson, S. E. Subsistence ecology and play among the Okavango Delta peoples of Botswana. *Hum. Nat.* **15**, 63–81 (2004).
42. Froehle, A. W. *et al.* Physical activity and time budgets of Hadza forager children: Implications for self-provisioning and the ontogeny of the sexual division of labor. *Am. J. Hum. Biol.* **31**, e23209 (2019).
43. Koster, J. & McElreath, R. Multinomial analysis of behavior: statistical methods. *Behav. Ecol. Sociobiol.* **71**, 1–14 (2017).
44. Bock, J., Johnson, S. E., Ecology, S., The, A. & Delta, O. Subsistence Ecology and Play among the Okavango Delta Peoples of Botswana. *Hum. Nat.* **15**, 63–81 (2004).
45. Harris, I., Osborn, T. J., Jones, P. & Lister, D. Version 4 of the CRU TS monthly high-resolution gridded multivariate climate dataset. *Sci. Data* **7**, 1–18 (2020).
46. Hijmans, R. J. *et al. Package 'raster' Geographic Data Analysis and Modeling*. https://github.com/rspatial/raster/issues/ (2020).
47. R Core Team. R: A language and environment for statistical computing. (2013).
48. Hijmans, R., Phillips, S., Leathwick, J. & Elith, J. dismo: Species distribution modeling, R package version 1.0–15 edn. (2016).
49. O'donnell, M. S. & Ignizio, D. A. *Bioclimatic Predictors for Supporting Ecological Applications in the Conterminous United States Data Series 691*. http://www.usgs.gov/pubprod (2012).
50. Longbottom, J. *et al.* Vulnerability to snakebite envenoming: a global mapping of hotspots. *Lancet* **392**, 673–684 (2018).
51. Jones, K. E. *et al.* PanTHERIA: a species-level database of life history, ecology, and geography of extant and recently extinct mammals. *Ecology* **90**, 2648–2648 (2009).
